# Chromatin remodeling-driven autophagy activation induces cisplatin resistance in oral squamous cell carcinoma

**DOI:** 10.1038/s41419-024-06975-1

**Published:** 2024-08-13

**Authors:** Su Young Oh, Jinkyung Kim, Kah Young Lee, Heon-Jin Lee, Tae-Geon Kwon, Jin-Wook Kim, Sung-Tak Lee, Dae-Geon Kim, So-Young Choi, Su-Hyung Hong

**Affiliations:** 1 Department of Microbiology and Immunology, School of Dentistry, Kyungpook National University, Daegu, South Korea; 2 Department of Oral and Maxillofacial Surgery, School of Dentistry, Kyungpook National University, Daegu, South Korea

**Keywords:** Oral cancer, Predictive markers

## Abstract

It is still challenging to predict the efficacy of cisplatin-based therapy, particularly in relation to the activation of macroautophagy/autophagy in oral squamous cell carcinoma (OSCC). We studied the effect of selected chromatin remodeling genes on the cisplatin resistance and their interplay with autophagy in 3-dimensional tumor model and xenografts. We analyzed gene expression patterns in the cisplatin-sensitive UMSCC1, and a paired cisplatin-resistant UM-Cis cells. Many histone protein gene clusters involved in nucleosome assembly showed significant difference of expression. Gain- and loss-of-function analyses revealed an inverse correlation between cisplatin resistance and *HIST1H3D* expression, while a positive correlation was observed with *HIST3H2A* or *HIST3H2B* expression. In UM-Cis, *HIST3H2A*- and *HIST3H2B*-mediated chromatin remodeling upregulates autophagy status, which results in cisplatin resistance. Additionally, knockdown of *HIST3H2A* or *HIST3H2B* downregulated autophagy-activating genes *via* chromatin compaction of their promoter regions. *MiTF*, one of the key autophagy regulators upregulated in UM-Cis, negatively regulated transcription of *HIST1H3D*, suggesting an interplay between chromatin remodeling-dependent cisplatin resistance and autophagy. On comparing the staining intensity between cisplatin-sensitive and –insensitive tissues from OSCC patients, protein expression pattern of the selected histone protein genes were matched with the in vitro data. By examining the relationship between autophagy and chromatin remodeling genes, we identified a set of candidate genes with potential use as markers predicting chemoresistance in OSCC biopsy samples.

## Introduction

Oral squamous cell carcinoma (OSCC) is the most common type of head and neck squamous cell carcinoma (HNSCC). Despite recent advances in OSCC treatments, the 5-year survival rate remains between 50–60% without substantial improvement [1]. The demand for chemoradiation has increased due to the aging population and longer life expectancy. However, its application is constrained by the presence of complications or toxicity [2, 3]. One of the main reasons for the limited effectiveness of chemotherapy is drug resistance, which contributes significantly to cancer recurrence and is a leading cause of death in cancer patients [4]. Platinum-based chemotherapy has been used to treat many solid tumors [5–8]. Cisplatin, one of the most common platinum compounds, interacts with DNA to form crosslinked DNA adducts, which trigger a series of intracellular events culminating in cell death [9, 10]. Cisplatin alone or in combination with other compounds is used in chemotherapy regimens, including first-line treatments of several cancer types, in approximately 50% of cancer patients [11]. However, the effectiveness of cisplatin is often hindered by the development of drug resistance in treating tumors, including OSCC [11]. To understand the mechanisms behind this resistance, researchers often generate cisplatin-resistant cell lines from parental cancer cells and compare their gene expression profiles.

One of the mechanisms by which tumor cells develop cisplatin resistance is through cytoprotective macroautophagy/autophagy. Autophagy can be upregulated in response to a range of stresses, including chemotherapy. Although autophagy is considered a protective mechanism for cisplatin-resistant phenotypes, the effect of autophagy on chemotherapy is context-dependent based on the tumor microenvironment [12–14]. The regulation of autophagy by specific histone proteins in tumor cells’ cisplatin resistances remains poorly understood. Together with transcription factors, chromatin remodeling due to fluctuations of histone proteins fine-tunes the transcriptional regulation of gene expression by making gene loci accessible to or hidden from the transcription machinery ultimately determining cell fate [15, 16]. Therefore, it is necessary to evaluate whether the perturbation of histone proteins leads to changes in biological pathways involved in cisplatin resistance in cancer cells.

We established a cisplatin-resistant cell line from the UMSCC1, one of the representative oral squamous cancer cells, followed by DNA microarray analysis. Significant changes in expression were observed in numerous genes related to nucleosome assembly. While extensive studies have explored drug responses in vitro, translating those findings to a clinical context is challenging [17]. We validated cisplatin efficacy in OSCC patients, categorizing them into two groups: cisplatin-sensitive and -insensitive. We then compared the expression profiles of candidate genes in tissue samples from these two groups. We specifically examined how changes in core histone proteins influenced chromatin assembly and induced autophagy in OSCC cells, in the context of cisplatin resistance. Our overall goal was to provide additional input that can be utilized in strategies to improve cisplatin efficacy in patients with OSCC.

## Materials and methods

### Two-dimensional (2D) and three-dimensional (3D) cell culture

OSCC cells were cultured in DMEM containing 10% FBS and 1% penicillin-streptomycin solution, at 37 °C in a 5% carbon dioxide (CO_2_) humidified atmosphere. The cell lines were tested for contamination every two months using the BioMycoX^®^ Mycoplasma PCR detection kit (CellSafe, D-100). For 3D spheroid formation, cells were seeded into a 96-well U-bottom ultra-low attachment plate (Corning Incorporated, 7007; 4000 cells/well) and cultured for 2–3 days to form spheroids with uniform sizes in each well ( > 300 μm in diameter). The spheroid size was determined by measuring the surface area of each group containing 6–8 spheroids using a Cell^3^iMager scanner CC-5000 (SCREEN Holdings Co., Ltd. Kyoto, Japan). Each spheroid’s surface area was the same at the beginning of the experiments, within the 5% error range.

### Organoid culture

Organoid culture was performed using mouse xenografts derived from UMSCC1, UM-Cis, or FaDu cells. Tongue tissue from patients with OSCC was also used for organoid culture. Tissue processing and organoid culture were performed as described by Driehuis et al. [18, 19]. More detailed information is provided in the Supplementary methods.

### Mouse xenograft model

We compared cisplatin efficacy in xenograft mice (6-week-old male BALB/c; Hyochang Science, Daegu, Korea). Cancer cells (2 × 10^6^/100 µL DMEM) from each cell line were injected subcutaneously into the right and left dorsal sides of mice. After 27 days, tumor formation was observed, and cisplatin was injected for 4 weeks. Detailed information is presented in the Supplementary methods.

### Chromatin accessibility assay

To evaluate nucleosome remodeling between UMSCC1 and UM-Cis cells, chromatin compaction was compared using the EpiQuik™ Chromatin Accessibility Assay Kit (Epigentek, P-1047-48). Changes in chromatin structure were identified by the degree of the Ct shift between nuclease-treated and untreated samples, following qPCR [20]. Heterochromatin-like compact DNA is not accessible to nucleases, resulting in only a slight shift of Ct between the digested sample and the undigested control, whereas decondensed DNA is accessible to nucleases, which is typically reflected by a larger Ct shift between experimental and control samples. Detailed information is presented in the Supplementary methods.

### Ethics approval and consent to participate

Human tissue specimens were used after receiving written informed consent from the patients, with approval from the Institutional Research Ethics Committee of Kyungpook National University Hospital (KNUH201704011) and adherence to the principles of the Declaration of Helsinki. All experimental protocols with mice followed the ARRIVE guidelines (Animal Research: Reporting of In Vivo Experiments) and were approved by the Animal Ethics Committee of Kyungpook National University (2017-94-2).

## Results

### Characterization of cisplatin-resistant UM-Cis cells

The proliferation rate of UM-Cis was decreased compared to that of UMSCC1 (Fig. S1A). Incubation with cisplatin resulted in significantly increased viability or decreased apoptosis of UM-Cis cells relative to UMSCC1 (Fig. S1B). In 3D spheroids, the size was significantly decreased with cisplatin treatment relative to a vehicle control in UMSCC1. However, no similarly significant decrease in spheroid size was observed in UM-Cis (Fig. S1C). Mouse xenografts derived from UM-Cis displayed significant resistance to cisplatin compared to UMSCC1-derived tumors (Fig. S1D). Representative hematoxylin and eosin (H&E) staining images of tumor tissues are shown in Fig. S1E. Both tumor tissues showed strong reactivity with the *KRT13* antibody, confirming their squamous epithelial origin.

### DNA microarray analysis and gene ontology enrichment

Following quantile normalization of raw DNA microarray data, the expression profile of UMSCC1 and UM-Cis cells was obtained and submitted to the Gene Expression Omnibus repository (GSE197561). A total of 797 differentially expressed genes (526 upregulated and 271 downregulated) were obtained between the two groups, using an average fold change of ≥ 2-fold and a *p*-value < 0.05 as our selection criteria. Shown in Fig. S2A is the heatmap using the z-score normalized data for genes with significant difference in expression > 4-fold. The 797 gene list was uploaded to the online software DAVID for biological process and cellular compartment analyses. According to biological process enrichment, nucleosome assembly was the most significant functional annotation term (*p* < 0.0001; gene count, 16; Fig. S2B). Telomere organization, which is closely related to nucleosome assembly, was the third most important pathway in biological processes (*p* < 0.0001; gene count, 7; Fig. S2B). In addition, GO cellular compartment enrichment revealed terms related to the nucleosome to be the most significantly different (Fig. S2C). As the role of nucleosome assembly in the mechanism of cisplatin resistance is not well understood, we focused on examining this biological process further within the context of OSCC.

### Differential expression of nucleosome assembly-related genes between UMSCC1 and UM-Cis cells

**Fig. 1:**
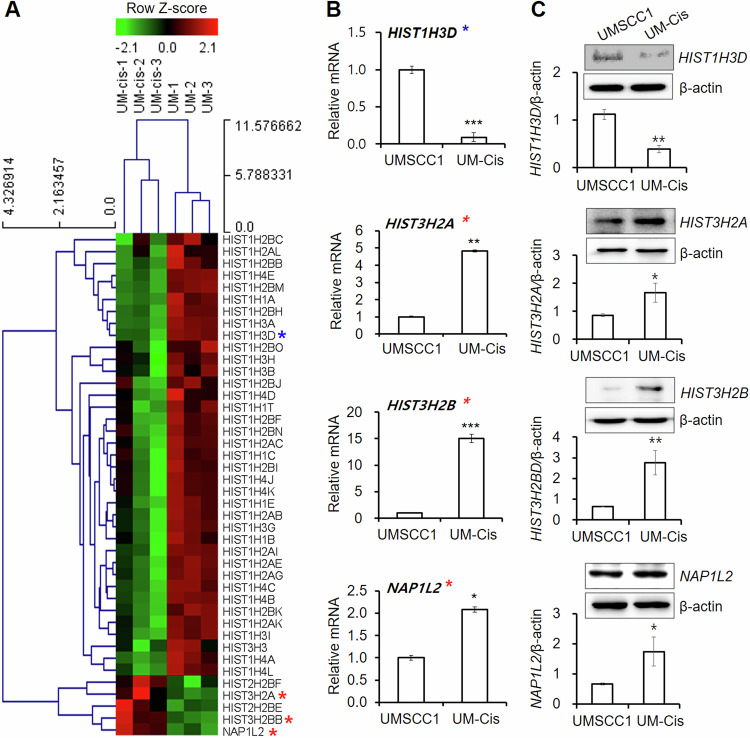
Gene expression of nucleosome assembly-related genes. **A** Heatmap for nucleosome assembly-related genes in the two groups with row *z*-score normalization (Fold change > 2, *p* < 0.05). The asterisks indicate representative genes consistent with the qPCR analysis in UMSCC1 and UM-Cis cells (Red asterisks denote increased expression in UM-Cis cells; blue asterisks denote decreased expression in UM-Cis cells). **B** The mRNA expression level of the nucleosome assembly-related genes. **C** Protein expression at the same samples. Results represent the mean ± standard deviation of three independent experiments (*p < 0.05, **p < 0.01, ***p < 0.001).

We identified several nucleosome assembly-related genes with >2-fold significant difference in expression (Fig. 1A). Of these, 14 genes were downregulated, while 28 genes were upregulated in UM-Cis. The mRNA and protein expression patterns of four genes: H3 clustered histone-4 (*H3C4*, also known as *HIST1H3D*), H2A clustered histone-25 (*H2AC25*, also known as *HIST3H2A*), H2B clustered histone-26 (*H2BC26*, also known as *HIST3H2B* and *HIST3H2BB*), and nucleosome assembly protein 1-like-2 (*NAP1L2*) were consistent with the microarray data (red and blue asterisks represent increased or decreased expression in UM-Cis, respectively; Fig. 1B, C).

### Effect of candidate gene knockdown on cisplatin efficacy in OSCC spheroids

*siHIST1H3D* pretreatment in UMSCC1 and FaDu spheroids led to an observable increase in cisplatin tolerance (Fig. S3A), and reduced *HIST1H3D* mRNA levels in both spheroids for 14 days (Fig. S3B). On the contrary, the knockdown of *HIST3H2A* or *HIST3H2B* was associated with a concomitant reduction in cisplatin resistance for UM-Cis spheroids (Fig. S3C). The knockdown effect of the siRNA was sustained for 14 days in both cisplatin or vehicle control-treated groups (Fig. S3D). The effect of these siRNAs on cisplatin efficacy were more pronounced in spheroids than in 2D cell cultures.

### Inverse correlation of cisplatin resistance and HIST1H3D expression level in organoids and xenografts

**Fig. 2:**
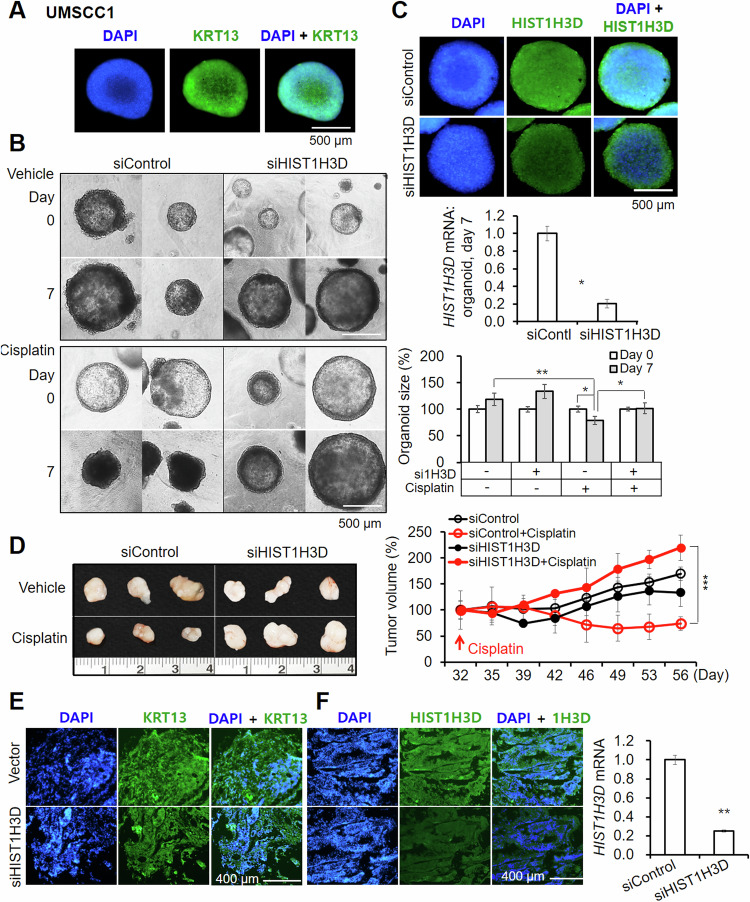
Effect of HIST1H3D knockdown on the chemosensitivity in organoids and mouse xenografts derived from UMSCC1. **A** IF staining in fixed organoids using an anti-KTR13 antibody, a representative squamous epithelial marker. **B** Organoids pretreated with siHIST1H3D or control siRNA, followed by cisplatin treatment for seven days. **C** siHIST1H3D efficiency in organoids, evaluated by IF staining and qPCR. Results represent the mean ± standard deviation of three independent experiments (*p < 0.05, **p < 0.01). **D** Cisplatin efficacy in mice xenografts derived from UMSCC1 spheroids transfected with siHIST1H3D or control siRNA. Tumor volume was measured till sacrifice (***p < 0.0005). **E** IF staining of mice tumor tissues with anti-KRT13 antibody. **F** Protein and mRNA expression of HIST1H3D in xenografts showing the efficiency of siHIST1H3D in xenografts (**p < 0.005).

We confirmed the organoids’ tumor identity using immunostaining against *KRT13* (Fig. 2A). The *siHIST1H3D*-transfected UMSCC1 organoids displayed increased tolerance to cisplatin compared to the control siRNA (Fig. 2B). *siHIST1H3D*-mediated gene knockdown effectively persisted for up to seven days in all cislpatin- or vehicle-treated organoids (Fig. 2C). Mouse xenografts derived from *siHIST1H3D*-transfected UMSCC1 spheroids showed signifcant resistance to cisplatin (Fig. 2D). Anti-KRT13 immunostaining in xenografts is shown in Fig. 2E. The knockdown effect of *HIST1H3D* in xenografts was maintained in the extracted tumor tissues (Fig. 2F). We repeated this experiment with organoids cultured from tongue OSCC tissues of patients and FaDu cell-derived xenografts. These organoids showed strong signal with anti-KRT13 antibody (Fig. S4A). Pretreatment with *siHIST1H3D* and cisplatin for seven days led to a significant increase in drug tolerance in both organoids (Fig. S4B), similar to UMSCC1 observations. The knockdown effect of the siRNA was sustained for seven days in both cisplatin or vehicle control-treated groups (Fig. S4C, D).

**Fig. 3:**
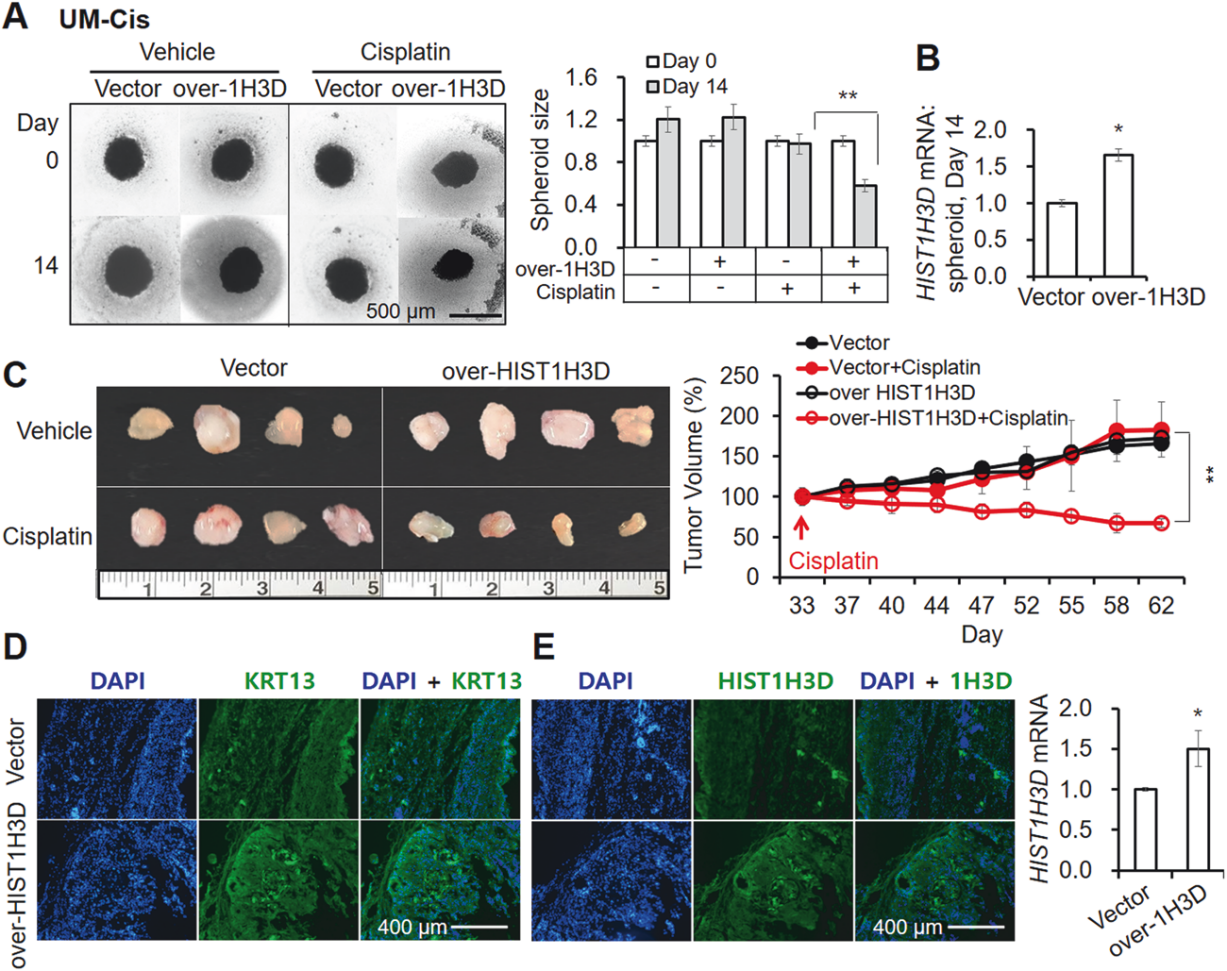
Effect of HIST1H3D overexpression on the chemosensitivity in spheroids and mouse xenografts derived from UM-Cis. **A** UM-Cis spheroids transfected with HIST1H3D-overexpression vector or control vector, followed by cisplatin treatment for 14 days. **B** mRNA expression level in spheroids transfected with HIST1H3D-overexpression vector for 14 days. Results represent the mean ± standard deviation of three experiments (*p < 0.05, **p < 0.005). **C** Cisplatin efficacy in mice xenografts derived from UM-Cis spheroids transfected with HIST1H3D-overexpression vector or control vector. Tumor volume was measured till sacrifice. **D** IF staining of mice tumor tissues with anti-KRT13 antibody. **E** Protein and mRNA expression of HIST1H3D in xenografts showing the efficiency of HIST1H3D-overexpression vector (*p < 0.05).

In UM-Cis spheroids, *HIST1H3D*-overexpression vector efficiency was maintained for 14 days (Fig. 3B), during which, cisplatin treatment resulted in a significant decrease of spheroid size, in comparison to the control vector group (Fig. 3A). Similarly, mouse xenografts developed from *HIST1H3D-*overexpressing UM-Cis spheroids markedly decreased in size compared to control vector group with cisplatin treatment (Fig. 3C). Anti-KRT13 immunostaining in xenografts is shown in Fig. 3D. The overexpression effect of *HIST1H3D* in xenografts was maintained in the extracted tumor tissues in both cisplatin or vehicle control-treated groups (Fig. 3E). These results suggest that the mechanism of cisplatin resistance is sensitive to fluctuations in *HIST1H3D* activity.

### Correlation between protein expression of candidate genes and cisplatin sensitivity in clinical OSCC tissues

**Fig. 4:**
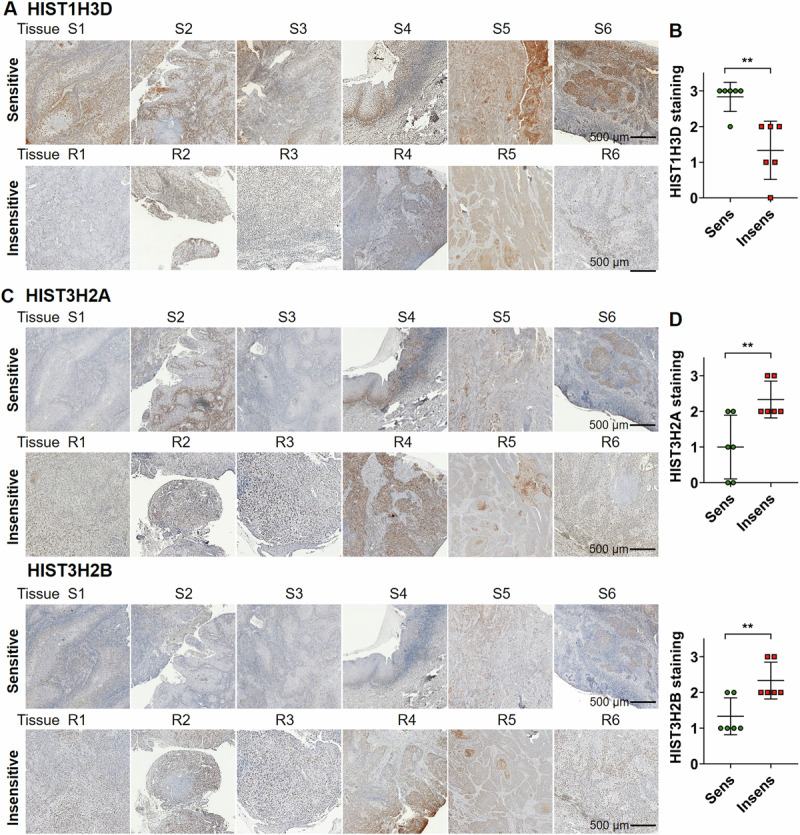
IHC analysis of OSCC tissues from patients for candidate protein genes. **A**, **C** Tissues from patients with OSCC showing cisplatin-sensitive and cisplatin-insensitive tissues were stained with anti-HIST1H3D, anti-HIST3H2A, and anti-HIST3H2B antibodies. **B**, **D** The level of protein expression on each specimen was scored as 0, 1, 2, and 3 (0 = negative, 1 = weak, 2 = intermediate, and 3 = strong) according to its staining intensity. **p < 0.01.

We investigated the relationship between *HIST1H3D*, *HIST3H2A*, and *HIST3H2B* protein expression and cisplatin sensitivity in patients with OSCC. Patients were classified as either sensitive or insensitive to cisplatin monotherapy based on changes in tumor size and survival. Figure 4A displays staining results from six tissue samples in each group. On comparing the staining intensity, tissues from cisplatin-sensitive patients exhibited a robust signal for *HIST1H3D*, whereas tissues from cisplatin-resistant patients displayed a comparatively weaker response (Fig. 4B). Conversely, the expression patterns of *HIST3H2A* and *HIST3H2B* were opposite, with higher levels in the cisplatin-resistant group (Fig. 4C, D). These results affirm the positive correlation between *HIST1H3D* expression, and the negative correlation between *HIST3H2A* and *HIST3H2B* expression and cisplatin sensitivity in OSCC tissues, supporting our findings.

Kaplan–Meier analysis revealed that high *HIST1H3D* expression was strongly associated with longer patient survival, while higher levels of *HIST3H2A* or *HIST3H2B* were linked to shorter survival (Fig. S5A). Our results regarding the mRNA expression of the three candidate genes in patient-derived tissues might be indirectly associated with post-treatment survival in OSCC patients. Disease-free survival analysis revealed no significant difference in the survival rates between the groups with high and low expression of the candidate genes (Fig. S5B).

### *HIST1H3D* is a target of transcriptional downregulation by *MiTF*

**Fig. 5:**
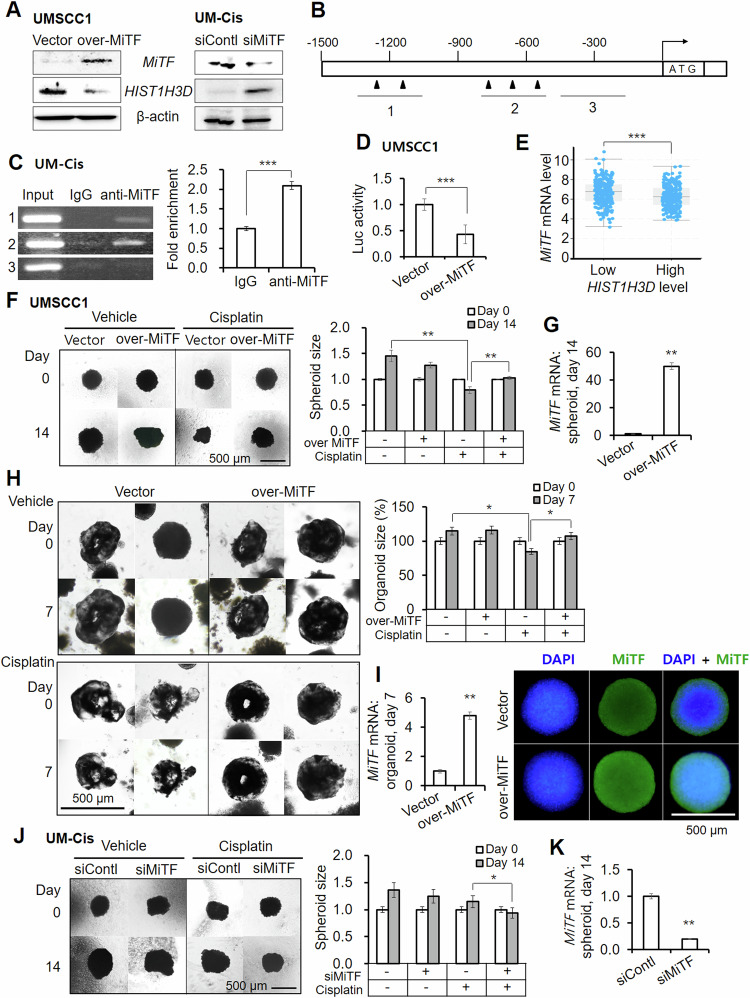
MiTF as a negative transcription regulator of HIST1H3D. **A** Protein expression in cells following exogenous overexpression or knockdown of MiTF in UMSCC1 or UM-Cis cells, respectively. **B** Schematic representation of the promoter regions of the HIST1H3D gene showing the consensus MiTF binding sites for ChIP analysis (black arrowheads in 1 and 2). **C** Gel electrophoresis image of PCR products from Chromatin IP using a MiTF antibody. Fold enrichment of DNA sequences that are associated with the target protein by qPCR. **D** Dual luciferase activity shows the direct binding of the 3′ regions of HIST1H3D gene and MiTF protein in UMSCC1 cells. **E** MiTF mRNA level in relation to HIST1H3D expression levels in HNSCC patient tissues. The data were obtained using the TCGA dataset through cBioportal. **F** UMSCC1 spheroids transfected with MiTF-overexpression vector, followed by cisplatin treatment. **G** MiTF-overexpression vector efficiency in spheroids after 14 days. **H** UMSCC1 organoids transfected with MiTF-overexpression vector, followed by cisplatin treatment. **I** mRNA and protein expression of MiTF in organoids showing the efficiency of MiTF-overexpression vector. **J** UM-Cis spheroids pretreated with siMiTF, followed by cisplatin treatment. **K** MiTF overexpression efficiency in spheroids after 14 days. Results represent the mean ± standard deviation of three independent experiments. (*p < 0.05, **p < 0.01, ***p < 0.005).

Our search for candidate transcription factors regulating *HIST1H3D* identified microphthalmia-associated transcription factor (*MiTF*) as a key regulator. We induced siRNA-mediated knockdown or vector-assisted overexpression of *MiTF* in UM-Cis, and UMSCC1/FaDu cells, respectively, followed by *HIST1H3D* protein expression analysis. In both cases, *MiTF* protein expression was consistently inversely related to *HIST1H3D* level, suggesting that *MiTF* might negatively regulate *HIST1H3D* (Figs. 5A, S6A). We investigated chromatin immunoprecipitation (ChIP) using UM-Cis cells expressing high levels of enodgenous *MiTF*. A schematic of the promoter region containing the consensus *MiTF*-binding sites is shown in Fig. 5B. The *MiTF-*bound *HIST1H3D* promoter sequences increased significantly compared to IgG control (Fig. 5C). Following luciferase reporter assay in UMSCC1 cells transfected with an *MiTF*-overexpression vector, luciferase activity from the *HIST1H3D* promoter region significantly decreased under *MiTF* overexpression (Fig. 5D). We conducted an analysis of the TCGA dataset using cBioPortal to validate the relationship between *HIST1H3D* and *MiTF* expression in tissues from HNSCC patients. As depicted in Fig. 5E, there was an inverse correlation between *MiTF* mRNA levels and *HIST1H3D* expression, suggesting that *MiTF* would function as a negative regulator of *HIST1H3D*. Together our findings suggest that *MiTF* could bind directly to the *HIST1H3D* promoter and negatively regulate the expression of *HIST1H3D*.

Regarding cisplatin resistance, *MiTF* overexpression in spheroids and organoids derived from UMSCC1 (Fig. 5F, H) and FaDu (Fig. S6B, D) significantly increasd the tolerance of cisplatin. Conversely, *siMiTF* pretreatment in UM-Cis spheroids reduced cisplatin resistance (Fig. 5J). Knockdown or overexpression efficiency of *MiTF* is shown in Fig. 5G, I and K; Fig. S6C, E.

### Interplay between histone protein expression and autophagy

**Fig. 6:**
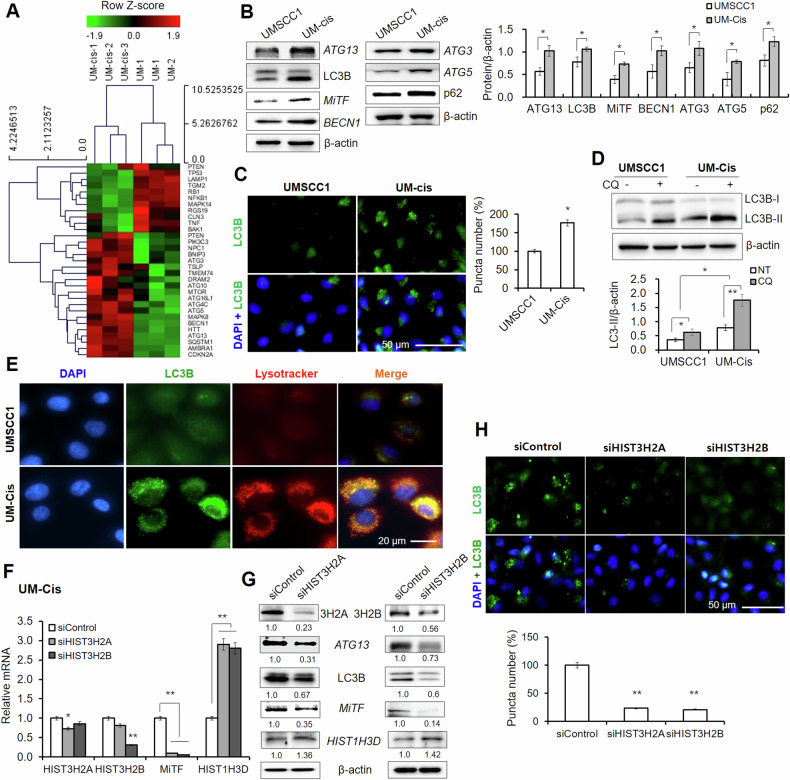
Interplay between histone proteins and autophagy. **A** Heatmap of genes involved in autophagy from DNA microarray dataset. **B** Autophagy-related proteins expression in UMSCC1 and UM-Cis cells. **C** LC3B puncta in both cells. **D** Comparison of autophagy flux under CQ treatment by LC3B protein expression level. **E** Representative images of co-localization of LC3B with lysosome using LysoTrackerTM Deep Red. **F**, **G** mRNA and protein expression in UM-Cis cells transfected with *siHIST3H2A* or *siHIST3H2B*. **H** LC3B puncta in UM-Cis cells transfected with *siHIST3H2A* or *siHIST3H2B*. Results represent the mean ± standard deviation of three independent experiments. (*p < 0.05, **p < 0.01).

As *MiTF* is a key regulator of autophagy induction, we explored the potential interplay between autophagy and histone proteins in the context of cisplatin resistance. A heatmap of autophagy-associated genes with > 2-fold significant difference is shown in Fig. 6A. Several genes involved in autophagy initiation, such as autophagy-related (*ATG*) were upregulated in UM-Cis. Protein levels of autophagy markers were higher in UM-Cis (Fig. 6B). UM-Cis displayed a pronounced distribution of cytoplasmic microtubule-associated proteins 1 A/1B light chain 3B (*MAP1LC3B*; abbreviated to LC3B) in punctate formations (Fig. 6C). We treated chloroquine (CQ), an autophagic flux inhibitor in both cells. Lipidated LC3B (LC3B-II) was increased in both CQ-treated cells. However, LC3B-II levels in UM-Cis were consistently higher than UMSCC1 in both baseline and CQ-treated conditions (Fig. 6D). Furthermore, LC3B-lysosome co-localization, a key marker of autophagy activation, was significantly higher in UM-Cis cells compared to UMSCC1 (Fig. 6E). Knockdown of *HIST3H2A* or *HIST3H2B* in UM-Cis resulted in downregulated *MiTF* mRNA with a consequent increase in *HIST1H3D* mRNA (Fig. 6F). This knockdown also led to lower levels of *ATG13*, *LC3B*, and *MiTF* proteins, while increasing *HIST1H3D* expression (Fig. 6G). The significant decrease of LC3B puncta levels under these conditions was also recorded in UM-Cis (Fig. 6H). These findings suggest that alteration of histone levels regulated autophagy-associated gene expression and subsequent autophagic activity in UM-Cis cells.

**Fig. 7:**
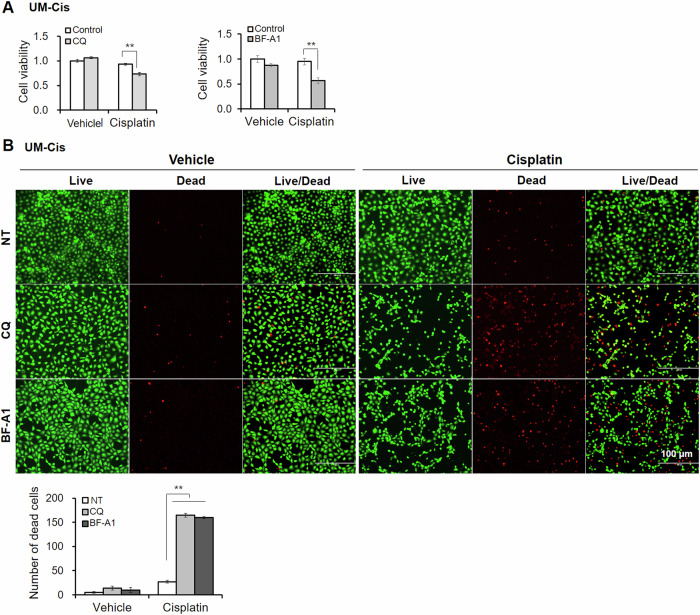
Effect of autophagy inhibitors on the cisplatin sensitivity in UM-Cis cells. **A** UM-Cis cell viability pretreated with autophagy inhibitors such as CQ or BF-A1, followed by cisplatin treatment. **B** LIVE/DEAD staining of UM-Cis cells pretreated with autophagy inhibitors. Dead cells were counted in each condition. Results represent the mean ± standard deviation of three experiments (**p < 0.01).

In UM-Cis, pretreatment with both CQ or a lysosomal inhibitor, bafilomycin A1 (BF-A1), resulted in comparable decreases in cisplatin resistance (Fig. 7A), supporting evidence for a link between autophagy activation and cisplatin resistance. LIVE/DEAD cell viability assay also showed that pretreatment with autophagy inhibitors reduced cisplatin resistance and increased cell death (Fig. 7B).

### Effects of histone proteins and autophagy on cisplatin resistance in YD-38/CIS cell line

Additional experiments were conducted with YD-38/CIS, a cisplatin-resistant cells derived from the parental OSCC cell line YD-38 (Fig. S7A). The expression patterns of *HIST1H3D*, *HIST3H2A*, and *HIST3H2B* in the two cell lines were consistent with those observed in UMSCC1 and UM-Cis (Fig. S7B). Moreover, pretreatment with either the *HIST1H3D*-overexpression vector or *siHIST3H2A* + *siHIST3H2B* in YD-38/CIS led to a reduction in cisplatin resistance (Fig. S7C). Additionally, autophagy was more activated in YD-38/CIS compared to YD-38 (Fig. S7D), and pretreatment with autophagy inhibitors resulted in reduced cisplatin resistance and increased cell death (Fig. S7E, F). These findings collectively suggest that *HIST1H3D*, *HIST3H2A*, and *HIST3H2B* genes might be involved in cisplatin resistance of YD-38/CIS along with autophagy.

### Chromatin structure modification

**Fig. 8:**
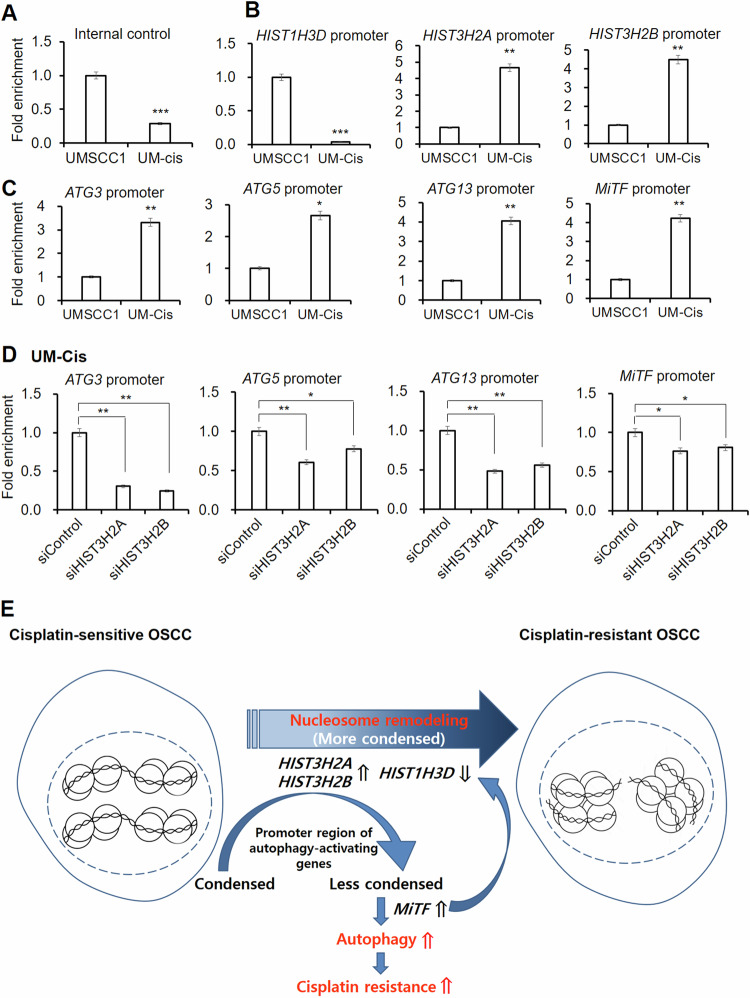
Chromatin assembly analysis in UMSCC1 and UM-Cis cells. **A**, **B** qPCR data showing the chromatin accessibility of specific genes’ region in UMSCC1 and UM-Cis cells. The lower C_t_ shift suggests more condensed chromatin conformations. **C** qPCR data showing the chromatin accessibility of autophagy-associated promoter regions. **D** qPCR data showing the chromatin accessibility of autophagy-associated promoter regions after transfection of UM-Cis cells with siHIST3H2A or siHIST3H2B. Results represent the mean ± standard deviation of three independent experiments (*p < 0.05, **p < 0.01, ***p < 0.005). **E** Schematic representation of the molecular mechanism exerting the cisplatin resistance in UM-Cis cells via the interplay between histone proteins and autophagy.

To further evaluate how protein level fluctuations of specific histones affect autophagy status, we analyzed the accessibility of chromatin due to histone-dependent chromatin remodeling in the UMSCC1 and UM-Cis. As shown in Fig. 8A, UM-Cis exhibited a lower Ct shift with internal control primers between nuclease-digested and undigested samples, suggesting more condensed chromatin structures. Next, the promoter region of *HIST1H3D* was significantly more condensed in UM-Cis than in UMSCC1 (Fig. 8B). In contrast, the promoters of *HIST3H2A* and *HIST3H2B* were less compact in UM-Cis, supporting the differential expression of these genes between the two cell lines. We examined the accessibility of the selected promoter regions of autophagy-facilitating genes for transcription. All tested gene promoter regions were less condensed in UM-Cis than in UMSCC1 (Fig. 8C), which may partially explain the increased expression of autophagy-promoting genes in UM-Cis. We repeated chromatin accessibility assay following treatment of UM-Cis with *siHIST3H2A* or *siHIST3H2B*. In the siRNA-transfected cells, the promoter regions of autophagy-promoting genes became significantly more condensed compared to control cells (Fig. 8D), indicating a possible downregulation of these genes following the knockdown of either *HIST3H2A* or *HIST3H2B*. Altogether, these results demonstrate that histone proteins may regulate autophagy-modulating genes at the transcriptional level, in the context of cisplatin resistance (Fig. 8E).

## Discussion

Numerous attempts are aimed at providing more effective chemotherapeutic regimens for cancer patients [21]. Previous data showed that several genes are associated with cisplatin resistance in OSCC cell-based experiments [22–26]. Nevertheless, there exists a substantial gap in correlating these gene expression patterns with drug response to predict chemotherapeutic efficacy in patients. Moreover, autophagy activation, a recognized mechanism contributing to cisplatin resistance in cancer, lacks comprehensive understanding within the context of OSCC. Our aim is to identify the mechanism of cisplatin resistance in OSCC and subsequently decipher the expression patterns of genes that can predict cisplatin efficacy in biopsy samples. This approach holds the potential to reduce unnecessary patient discomfort associated with drug side effects.

Chromatin conformation, in which histones play an important role, is essential for mediating various transcriptional responses that affect disease status, including cancer [27–34]. Kamur et al. suggest that understanding eukaryotic genomes requires looking beyond the 3D structure of chromatin fibers. They highlight the importance of examining 4D genomics, which considers how gene expression is regulated in response to environmental factors such as developmental stages, growth conditions, and diseases [35]. Alterations in chromatin assembly lead to perturbations in the expression of genes involved in intracellular functions such as autophagy, a primary pathway for cisplatin resistance [36–38]. Furthermore, the nucleus is a major regulator of autophagy through transcriptional and epigenetic modifications [39, 40]. Therefore, the nucleosome assembly status is likely involved in regulating drug resistance in cancer. In this study, many genes involved in nucleosome assembly exhibited significantly different expression patterns between cisplatin-sensitive and –insensitive cells. As the mechanisms of cisplatin resistance in the context of nucleosome assembly remain uncharacterized, we focused on further investigating this biological process.

Chromatin can exist in more compact (inaccessible) or relaxed (accessible) conformations, depending on the function being supported in the genome, either locally or globally. The H2A/H2B dimers contribute to nucleosome compaction, which results in decreased gene activity [41, 42]. Cisplatin-resistant cancer cells show significant changes in their transcription profile, as a result of epigenetic alterations including histone remodeling [37]. However, the link between changes in histone protein transcription levels and drug resistance in cancer is not well understood. In UM-Cis, *HIST3H2A* and *HIST3H2B* were upregulated, leading to a greater degree of global chromatin compaction compared to UMSCC1. However, the promoter regions of several autophagy-activating genes showed more relaxed chromatin structures, likely resulting in higher expression. Furthermore, knockdown of either *HIST3H2A* or *HIST3H2B* resulted in the promoter regions becoming more condensed. Collectively, the chromatin state in UM-Cis was altered by the changes in histone protein expression, and the promoter regions of autophagy genes were locally decondensed. Consequently, autophagy was upregulated and contributed to the development of cisplatin resistance in OSCC.

Unlike most histone protein-coding genes, *HIST1H3D* expression was significantly reduced in UM-Cis due to negative transcriptional regulation by *MiTF*. Vyas et al. suggested that *MiTF* activation plays a significant role in developing cisplatin resistance [43]. Our data support a strong relationship between specific histone proteins expression level and autophagy activation. The ultimate goal was to verify the results by tracking chemotherapy outcomes in patients who donated tissues for the trial. Specifically, we examined *HIST1H3D, HIST3H2A,* and *HIST3H2B* protein expression in cisplatin-sensitive and insensitive OSCC tissues. However, due to the small number of patients receiving cisplatin monotherapy, each group included only six tissue samples. Despite this limitation, the expression patterns of these histone protein genes in patient tissues strongly supported our findings. Moving forward, it is crucial to expand our research through retrospective studies, incorporating a larger number of patient tissues. Future studies will delve into the molecular mechanisms responsible for autophagy activation by these histone proteins, and explore potential associations with other cisplatin resistance-related genes. These investigations are crucial for understanding the mechanisms underlying cisplatin resistance that depends on histone proteins.

In conclusion, we demonstrated that chromatin assembly changes may be related to cisplatin resistance *via* autophagy regulation in OSCC. We identified that the mRNA or protein expression of *HIST3H2A, HIST3H2B*, and *HIST1H3D* genes may be used as indicators to predict, to some degree, the cisplatin efficacy in patients with OSCC. This may in turn lead to the design of chemotherapeutic regimens with improved efficiency, based on a more personalized treatment plan according to the unique genetic landscape of each patient.

### Supplementary information

Supplementary file

Western raw data

## Data Availability

The expression profile of UMSCC1 and UM-Cis was submitted to the Gene Expression Omnibus repository (GSE197561). The raw/processed data required to reproduce these findings are available with the corresponding author and can be given upon reasonable request.
