# Supplementary material for: Chromatin remodeling-driven autophagy activation induces cisplatin resistance in oral squamous cell carcinoma
Source: Cell Death Dis. 2024 Aug 13;15(8):589. doi: 10.1038/s41419-024-06975-1 (PMC11322550; doi:10.1038/s41419-024-06975-1)
Supplement: Supplementary file 1 — Supplementary file [file 41419_2024_6975_MOESM1_ESM.docx]

**Supplementary Methods, Figures, and Tables**

**Chromatin remodeling-driven autophagy activation induces cisplatin resistance in oral squamous cell carcinoma**

Su Young Oh, Jinkyung Kim, Kah Young Lee, Heon-Jin Lee, Tae-Geon Kwon, Jin-Wook Kim, [Sung-Tak Lee](https://www.sciencedirect.com/science/article/pii/S0022391318303688?via%3Dihub#!), [Dae-Geon Kim](https://pubmed.ncbi.nlm.nih.gov/?term=Kim+DG&cauthor_id=34361027), So-Young Choi, Su-Hyung Hong

**Supplementary Methods, Figures, and Tables contain:**

**Supplementary Methods**

**Supplementary Figures S1-7**

**Supplementary Tables S1-3**

**Supplementary Methods**

**Chemicals and reagents**

The UMSCC1 (mouth floor tumor) was obtained from Merck Millipore (Cat # SCC070, RRID: CVCL_7707, Darmstadt, Germany), and FaDu (hypopharyngeal tumor) was obtained from the American Type Culture Collection (ATCC, Cat # HTB-43, RRID: CVCL_1218). YD-38 (lower gingiva tumor) was purchase from Korean Cell Line Bank (Seoul, South Korea). YD-38/CIS was provided by professor Pil-Young Yun (Seoul National University Bundang Hospital, Department of Oral and Maxillofacial Surgery). Dulbecco’s Modified Eagle’s Medium (DMEM, Hyclone, SH30243.01), fetal bovine serum (FBS, Hyclone, SH30919.03), and penicillin-streptomycin were acquired from Invitrogen (15140-122; Carlsbad, CA, USA). Trizol was purchased from Sigma-Aldrich (15596-018; Merck, Darmstadt, Germany), and TOPreal™ SYBR Green qPCR PreMIX was purchased from Enzynomics (RT500S; Daejeon, South Korea). LIVE/DEAD staining kit (calcein-AM/propidium iodide (PI); C3099 and P3566, respectively), LysoTrackerTM Deep Red (L12492), Alexa Fluor 488 conjugate secondary antibody (A-11001), and Alexa Fluor 633 conjugate secondary antibody (A-21070) were purchased from Invitrogen (Carlsbad, CA, USA). Mouse anti-HIST1H3D (sc-134355), anti-MiTF (sc-515925, RRID:AB_2828036), anti-β-actin (sc-47778, RRID:AB_626632), anti-CDH2 (sc-8424, RRID:AB_626778), and anti-BECN1 (sc-48341, RRID:AB_626745) antibodies were from Santa Cruz Biotechnology, Inc (Santa Cruz, CA, USA). Anti-caspase 3 (9662, RRID:AB_331439), anti-PARP antibodies (9532, RRID:AB_659884), and anti-HIST3H2B (12364, RRID:AB_2714167) were purchased from CST (Cell Signaling Technology, MA, USA). Anti-CDH1 (610181, RRID:AB_397580) and anti-ALDH1A1 (611194, RRID:AB_2224312) antibodies were acquired from BD Biosciences. Anti-HIST3H2A (10445-1-AP, RRID:AB_2116701), anti-MAP1LC3B (14600-1-AP, RRID:AB_2137737), anti-KRT13 (10164-2-AP, RRID:AB_2134679), anti-ATG3 (11262-2-Ap, RRID:AB_2059234), anti-ATG5 (66744-1-Ig, RRID:AB_2882092), and anti-ATG13 (18258-1-AP, RRID:AB_2130658) antibodies were from Proteintech (Rosemont, IL, USA). Anti-NAP1L2 antibody was from Bioss Antibodies (bs-11172R). Cisplatin was purchased from Merck (1134357).

**Establishment of cisplatin-resistant cells from UMSCC1 cell line**

Cisplatin-resistant UM-Cis cells were derived from original UMSCC1 cells by continuous exposure to stepwise increasing cisplatin concentrations. Initially, the exponentially growing cells were exposed to a 1 μg/mL concentration of cisplatin in DMEM medium with 10% FBS and 1% penicillin/streptomycin, and further subcultured upon reaching 70–80% confluency. Each new subculture was supplemented with a higher cisplatin concentration which increased gradually, and the above process was repeated. Cell subline aliquots were saved and cryopreserved at each incremental concentration. The final cisplatin concentration used in the culture medium of UM-Cis cells was 5 μg/mL, which corresponded to the IC_50_ of the seeding UMSCC1 cells. In addition, a parental cell line treated with a vehicle solution of 0.1% (v/v) DMSO in PBS, was cultured in parallel during this period as a control cell line.

**Cell viability test**

Cisplatin sensitivity was determined using 2D MTT and 3D spheroid or organoid models derived from OSCC cells or primary tissues. To evaluate cell viability under 2D conditions, cells were seeded into 96-well plates at a concentration of 1 × 10^4^ cells/well. The following day, cells were treated with cisplatin in fresh media and incubated for an additional 48 h. We used DMSO (0.1% v/v in PBS) as a vehicle control. Cell viability was assessed by MTT assay, and absorbance was measured at 540 nm using an ELISA microplate reader (Molecular Devices, Sunnyvale, CA, USA). The effect of cisplatin on the 3D spheroid size (measured as surface area) was also evaluated. Spheroids were allowed to form in a 96-well U-bottom ultra-low attachment plate with uniform size in each well (> 300 μm in diameter). After carefully aspirating the medium, fresh medium containing cisplatin was added to each well. Spheroid growth was monitored over 14 days. Organoids grew to approximately 300 μm in diameter after about one month from the start of the culture. In the 24-well plates, the experiment was conducted with five organoids per well as a group, and the size of the organoids was measured after seven days using phase-contrast microscopy (5× magnification). The vehicle control used was DMSO 0.1% (v/v) in PBS.

For LIVE/DEAD staining, cells were cultured in 24-well plats for 24 h. Cells were treated with autophagy inhibitors for 2 h, followed by cisplatin treatment for another 24 h. After washing cells, they were stained with LIVE/DEAD staining kit (calcein-AM/propidium iodide (PI), according to the manufacturer's instructions. Fluorescent image was captured using ZEISS Axio microscope.

**Organoid culture**

Primary tissue pieces were washed with 45 mL of ice-cold Advanced DMEM/F12 medium (Thermo Fisher Scientific, 12634-010) supplemented with 1× GlutaMAX (Thermo Fisher Scientific, 35050-061), penicillin–streptomycin (Thermo Fisher Scientific, 15140-122), 10 mM HEPES (Thermo Fisher Scientific, 15630-056), and 100 μg/mL Primocin (Invivogen, ant-pm-1). The complete medium was termed Advanced DMEM (adDMEM/F12) +/+/+. To prevent contamination, 100 μg/mL Primocin (Invivogen, ant-pm-1) was added to the adDMEM/F12 +/+/+ medium. Tissue pieces were crushed into small fragments of 1–3 mm^3^ in a 10 cm cell culture dish using surgical scissors or scalpels. Minced tissue samples were digested for <1 h incubations in TrypLE (Gibco, 12605-028), and the contents were mixed every 10–15 min by vigorous shaking. When mixture became cloudy, the remaining tissue fragments became disrupted. We pipetted up and down 20 times using a P1000 pipette. After centrifugation at 200 × g for 5 min at 4°C, the pellets were resuspended in 10 mL of adDMEM/F12+++ medium and filtered using a 100 μm cell strainer. The samples were centrifuged one more time at 200 × g for 5 min at 4°C, the supernatant was aspirated, and pellets were resuspended in cold BME (R&D Systems, 3533-005-02). Approximately 10 μL droplets were plated on the bottom of preheated suspension culture plates (Greiner Bio-One, M9312). After seeding, the plates were inverted and incubated at 37°C for 30 min for BME to solidify. Pre-warmed organoid medium (adDMEM/F12+++ containing 1× B27 supplement (Thermo Fisher Scientific17504-044), 1.25 mM N-acetyl-l-cysteine (Sigma-Aldrich, A9165), 10 mM nicotinamide (Sigma-Aldrich, N0636), 50 ng/mL human EGF (PeproTech, AF-100-15), 0.5 μM A83-01, 10 ng/mL human FGF10 (PeproTech, 100-26), 5 ng/mL human FGF2 (PeproTechm, 100-18B), 1 μM prostaglandin E2 (Tocris Bioscience, 2296), 0.3 μM CHIR 99021 (Sigma-Aldrich, SML1046), 1 μM forskolin (R&D Systems, 1099), 4% R-spondin, and 4% Noggin (both produced *via* the r-PEX protein expression platform at U-Protein Express BV) was subsequently added to the plate. The medium was changed every 2–3 days, and organoids were split once every 1–2 weeks. Cisplatin efficacy was monitored for seven days using a Nikon ECLIPSE Ti microscope (Nikon Imaging Japan Inc., Tokyo, Japan).

**Mouse xenograft model**

The UMSCC1 and UM-Cis cell-treated groups consisted of four mice each. After tumor formation, mice within each group were randomly divided into two subgroups: cisplatin- or vehicle- injection control (total tumor-forming groups n=4). Cisplatin (2.5 mg/kg) or vehicle control was intraperitoneally injected two times each week and mice were sacrificed on the 29^th^ day following cisplatin administration. For efficacy assessment, tumor volume was measured using a caliper by an individual who was not involved in the experiment. Cases where tumors did not form were excluded from the analysis. To further evaluate the effect of candidate genes on cisplatin efficacy in mouse xenografts, we injected UMSCC1 spheroids (100 spheroids per injection, *approximately* 1 x 10^6^ cells/100 µL DMEM) previously transfected with control siRNA or *siHIST1H3D*, subcutaneously using a 22-gauge needle in the right- and left side of the back. UM-Cis spheroids transfected with empty vector or *HIST1H3D*-overexpression vector, respectively, were injected at the same condition. We used spheroids for this *in vivo* experiment owing to the improved gene knockdown or exogenous overexpression efficiency with transient transfection in 3D spheroids over cells from 2D cultures [1-3]. With regard to tumor size, we followed the relevant ARRIVE guidelines for sacrificing the animals when mouse weight was reduced by 20% or tumor volume was ≥10 cm^3^.

**DNA microarray analysis**

Microarray analysis was performed on six samples with each cell line represented in triplicate to measure the difference in gene expression levels between UMSCC1 and UM-Cis cells. We used the Affymetrix GeneChip® Human Gene 2.0 ST array (RRID:SCR_012825, Affymetrix Inc., Santa Clara, CA, US) containing a 40,716 gene-level probe set. Total RNA was obtained from UMSCC1 and UM-Cis cells cultured without cisplatin, using TRIzol™ as the extraction reagent. RNA quality was assessed using an Agilent 2100 Bioanalyzer (Agilent Technologies, Santa Clara, CA, USA), and quantity was determined using an ND-1000 spectrophotometer (NanoDrop Technologies, Wilmington, DE, USA). The RNA samples were prepared for the Affymetrix procedure per the recommended protocol (<http://www.affymetrix.com>). Total RNA from each sample was converted to double-stranded cDNA using a random hexamer primer incorporating a T7 promoter. Amplified RNA was generated from the double-stranded cDNA template *via* an *in vitro* transcription reaction and purified using the Affymetrix sample cleanup module. Thereafter, the cDNA was fragmented by the UDG and APE1 restriction endonucleases and end-labeled *via* a terminal transferase reaction incorporating a biotinylated dideoxynucleotide. Fragmented end-labeled cDNA was hybridized to the Affymetrix arrays and stained using streptavidin–phycoerythrin conjugate. The Affymetrix array was scanned using an Affymetrix Model 3000 G7 scanner, and the image data was extracted through the Affymetrix Command Console Software 1.1. Data mining and graphic visualization were performed using the ExDEGA software (Ebiogen, Seoul, Korea). The identified gene list depicting differential gene expression was uploaded to the online software DAVID for molecular function and biological process analyses, followed by gene clustering into pathways for hierarchical sorting.

**qPCR and protein expression analysis**

Quantitative PCR was performed for mRNA expression analysis. RNA extraction, cDNA synthesis, and gene expression normalization were performed according to standard protocols. The primers employed for qPCR are listed in Supplementary Table S1. The qPCR reaction was performed in triplicate wells per condition, using an ABI 7500 real-time PCR system (Applied Biosystems, Foster City, CA, USA). Gene expression levels were normalized to the levels was *GAPDH*. The fold change in gene expression was calculated based on the delta cycle to threshold (ΔCt) values, determined by normalizing the average Ct value of each sample to that of the endogenous *GAPDH* control and then calculating the 2^−ΔΔCt^ value for each treatment.

For protein expression analysis, total protein was extracted, and the concentration measured. Equal protein loading amounts (20–40 µg) were resolved using 8–10% sodium dodecyl sulfate-polyacrylamide gel electrophoresis (SDS-PAGE) and the proteins were transferred to nitrocellulose membranes. After blocking with 5% skim milk for 60 min, the membrane was incubated overnight at 4°C with the relevant primary antibodies; β-actin served as the loading control. Horse radish peroxidase (HRP)-conjugated secondary antibodies at 1:5000 dilutions were applied for 1 h at room temperature; the blot was washed three times in Tris-buffered saline containing 0.1% Tween 20. Protein bands were detected by enhanced chemiluminescence.

**Transfection of siRNA or overexpression vector**

The OSCC cells, 3D spheroids, and organoids were transfected with siRNA mixture comprising of 2–3 specific oligonucleotides (Santa Cruz Biotechnology). The cells ($1\times{10}^{4}$) were seeded on a 96-well plate, and on the next day, the medium was replaced with a serum-free medium immediately before transfection with specific or control siRNA at a final concentration of 10 nM, using Lipofectamine 3000 (Thermo Fisher Scientific, L3000015). After 24 h, cultures were treated with cisplatin for an additional two days, followed by an MTT cell viability assay. To evaluate the effect of specific siRNA on cisplatin sensitivity of 3D spheroids or organoids derived from OSCC cells or primary tissues, siRNA was used at a final concentration of 3 nM / 96 wells or 15 nM / 24 wells. After 24 h, vehicle control or cisplatin was added and cultures were monitored for an additional 7–14 days. The pCMV3-ORF-vector (Sino Biological, HG18977-UT) was used for exogenous overexpression of *HIST1H3D* or *MiTF* at a final concentration of 100 ng / 96 wells or 500 ng / 24 wells. The empty pCMV3-ORF vector was used as a vehicle control.

**Immunohistochemical analysis**

For the staining of human tissues or mouse xenografts, obtained sections were blocked for 5 min, followed by incubation for 2 h at room temperature with specific antibodies (1:500). The IHC staining was performed using the UltraTek HRP Anti-Polyvalent Kit (ScyTek Laboratories, AMF080). Tissues were counterstained with hematoxylin and eosin. Positive cells were quantified using ImageJ (RRID:SCR_003070).

Human tissue blocks were obtained from patients with OSCC who underwent biopsy or tumor resection for oral cancer treatment from 2016 to 2022 at the Kyungpook National University Hospital. Patient information is presented in Supplementary Table S2. After dewaxing, sections were blocked for 5 min, followed by incubation for 2 h at room temperature with specific primary antibodies (1:500-1:100). IHC staining was performed using the UltraTek Horseradish Peroxidase (HRP) Anti-olyvalent Kit (ScyTek Laboratories, USA); the chromogen used was 3,3-diaminobenzidine (Dako, USA). The image was acquired under light microscope at 40 × magnification. The protein level on each specimen was scored as 0, 1, 2, and 3 (0 = negative, 1 = weak, 2 = intermediate, and 3 = strong) according to its staining intensity.

**Chromatin immunoprecipitation (ChIP) and luciferase reporter assay**

A chromatin Immunoprecipitation Kit from EpiGentek (P-2002-1) was used for ChIP analysis. After crosslinking UM-Cis cells with paraformaldehyde for 20 min, the cell pellets were lysed in lysis buffer, followed by sonication to shear DNA to an average fragment size of 200–1000 bp. The DNA fragments were mixed with anti-MiTF or IgG antibody solutions and immunoprecipitated overnight at 4°C. The precipitated chromatin fragments were purified and analyzed *via* RT-qPCR and PCR.

For luciferase assay, the UMSCC1 cells were transfected with control or *MiTF*-overexpression vector. *HIST1H3D* promoter region-conjugated vector was co-transfected using Lipofectamine 3000 in triplicate wells. After 24 h incubation, a dual luciferase assay was conducted using the Dual-Luciferase Reporter Assay Kit (Promega, E1910). The assay, as well as the subsequent measuring of luciferase activity to evaluate the direct binding between the *HIST1H3D* gene and the *MiTF* protein, were performed according to the manufacturer's instructions. Firefly luciferase activity was normalized to the *Renilla* luciferase activity. The primers employed for PCR are listed in Supplementary Table S3.

**Immunofluorescence (IF) staining**

IF staining was performed for culture spheroids, organoids, and xenografts. Briefly, spheroids or organoids in Matrigel were released in PBS, followed by immediate fixation with 4% paraformaldehyde for 2 h and treatment with blocking solution (5% serum in 1× PBS plus 0.5% Triton-X100) for 1 h before incubation with the primary antibodies overnight. The next day, the organoids were washed in PBS-0.5% Triton-X100 three times, and exposed to the secondary antibody for 2 h. To counterstain for nuclei, the organoids were suspended in a drop of VECTASHIELD mounting medium with DAPI and loaded on a CoverWell Imaging Chamber for fluorescence microscopy.

To evaluate the co-localization of LC3B and lysosome, the cells were preincubated with LysoTrackerTM Deep Red for 1 h, followed by fixation with 4.0% paraformaldehyde for 5 min at room temperature. The fixed cells were then washed with PBS, and permeabilized with 1% Tween-20 in PBS for 5 min. The cells were incubated with 1% bovine serum albumin in PBS for 2 h and subsequently with anti-LC3 antibody overnight at 4°C, followed by Alexa Fluor 488 goat anti-rabbit IgG as a secondary antibody. Images were captured on a ZEISS Axio microscope (ZEISS Microscopy, Germany, Oberkochen).

**Chromatin accessibility assay**

Chromatin isolated from each cell line was treated with a nuclease mix, followed by DNA extraction. Briefly, UMSCC1 and UM-Cis cells were cultured up to 80% confluency and two aliquots of cells were lysed to isolate the chromatin. One aliquot was digested with a nuclease mix, while the other served as the undigested control (no nuclease present). After incubating at 37ºC for 4 min, the reaction was quenched with stop solution. Following DNA purification, qPCR was performed using internal control primers. Fold enrichment was calculated as the ratio of the amplification efficiency of DNA samples treated with nuclease, over that of non-nuclease-treated control samples. The primers employed for qPCR are listed in Table S3.

**Statistics**

All *in vitro* experiments were performed two or three times. Statistical parameters, including analysis of the *in vivo* results obtained from the murine models, are presented in the figure legends. All statistical analyses were conducted using the Origin v.8.0 (OriginLab, Northampton, MA, USA) and R software. One-way ANOVA and unpaired t-test were used for the statistical testing of comparisons between three or more, and two groups, respectively. The mRNA expression between cisplatin-sensitive and resistant tissue groups was compared using the Mann–Whitney U test. For survival analysis, we obtained the relevant dataset from cBIOPORTAL, specifically HNSCC (TCGA, Firehose Legacy). Subsequently, we conducted Kaplan-Meier survival analysis using MediCalc. The optimal cut-off values were determined using the ROC AUC curve with the R package. A *p*-value < 0.05 was considered as statistically significant. Significant *p*-values are shown in each figure.

**Supplementary Figures**

**
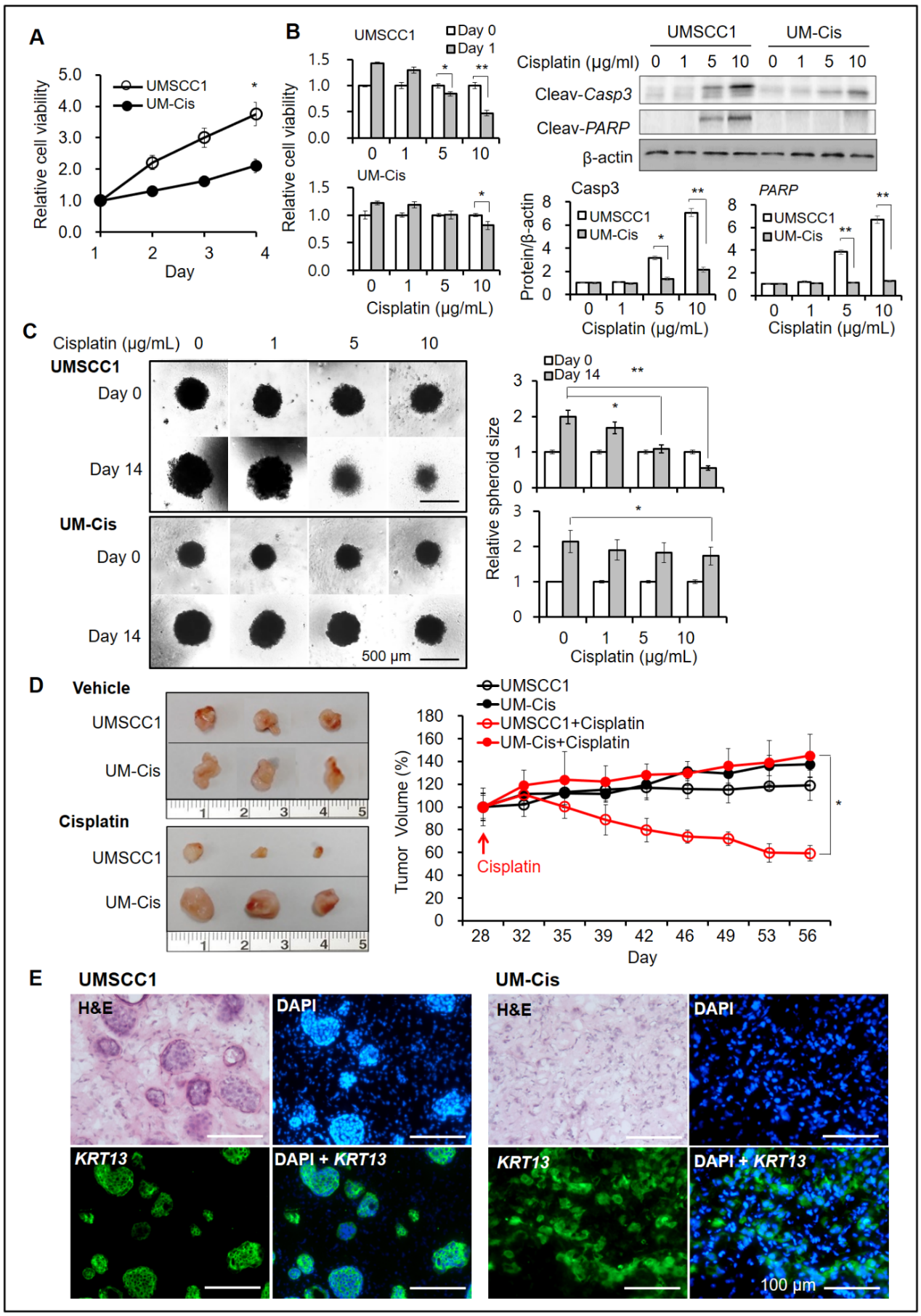
**

**Fig. S1 (related to Figure 1)**

**Comparison of cisplatin efficacy in UMSCC1 and UM-Cis cells.** **A** Growth rate by MTT assay. **B** Cell viability and apoptosis in both cell lines with cisplatin treatment. **C** Representative images of 3D spheroid and their growth with cisplatin treatment measured by the average surface area. Results represent the mean ± standard deviation of three independent experiments (*p <0.05, **p <0.01). **D** The effect of cisplatin on mice xenografts formed with UMSCC1 or UM-Cis cells (*p <0.01). **E** IF staining of mice tumor tissues using anti-KRT13 antibody, the representative marker of oral squamous cell differentiation.

**
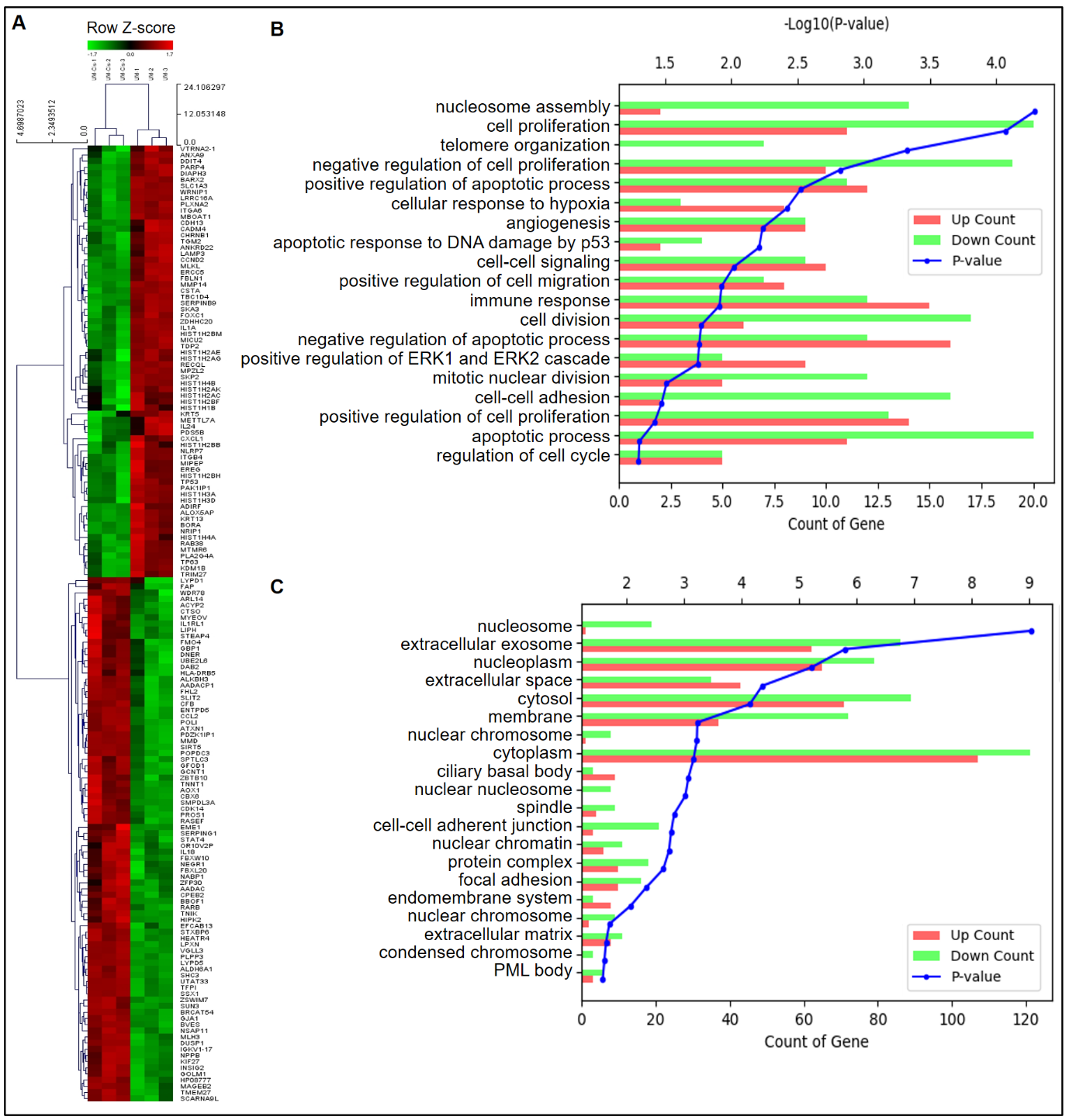
**

**Fig. S2 (related to Figure 1)**

**DNA microarray analysis of UMSCC1 and UM-Cis cell groups.** Microarray analysis with six samples, each cell line in triplicate. **A** Heatmap of differentially expressed genes among the matched groups with a fold change of >3 and p-value of <0.05. Functional annotation search with differentially expressed genes identified in a (**B**) specific biological process (GOTERM BP DIRECT) and (**C**) cellular compartment (GOTERM CC DIRECT).

**
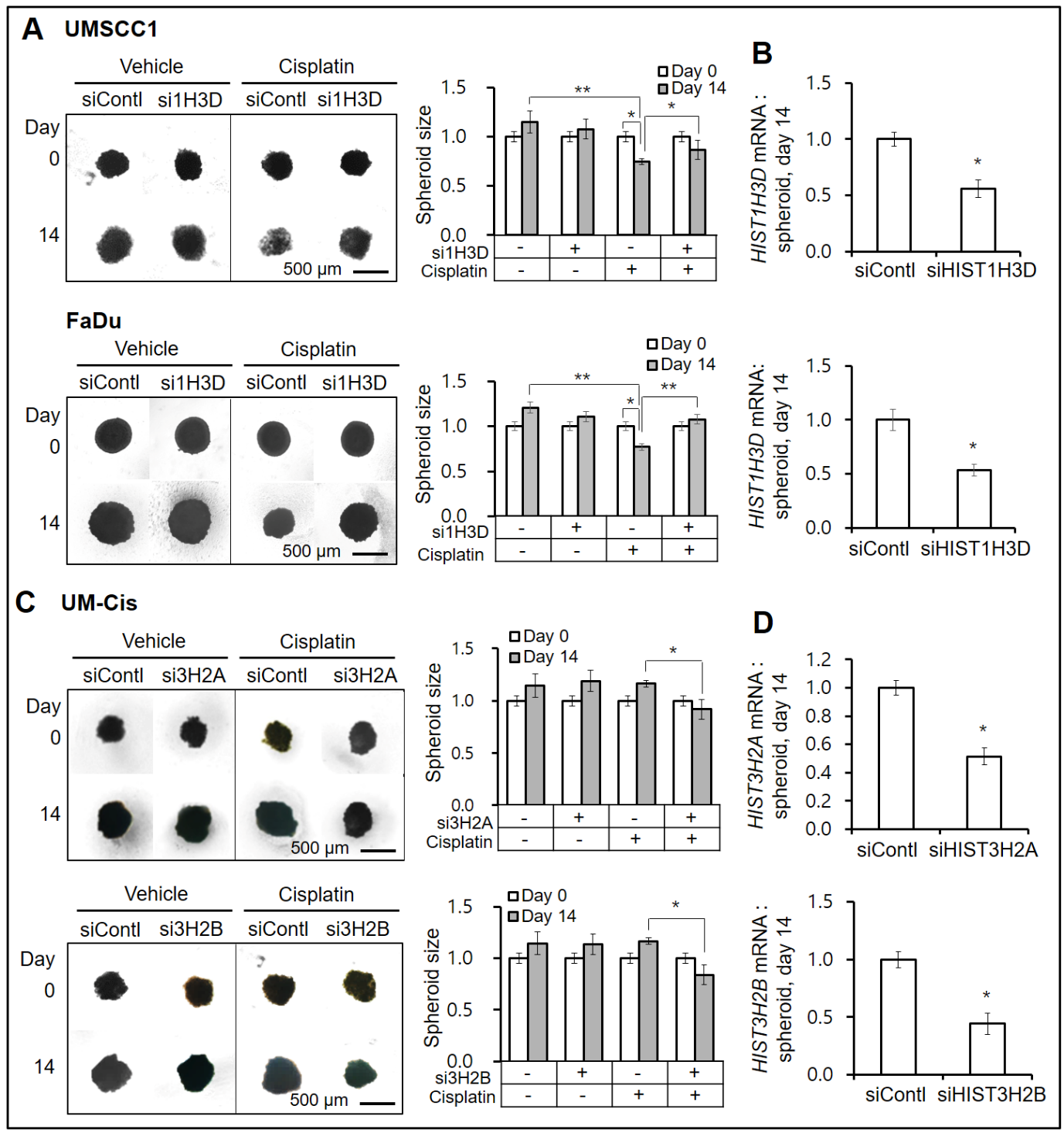
**

**Fig. S3 (related to Figure 1)**

**Effect of knockdown of the cisplatin resistance-related genes on the cisplatin efficacy in OSCC cells.** **A** Representative images of UMSCC1 spheroids pretreated with *siHIST1H3D*, followed by cisplatin treatment for 14 days. **B** UM-Cis spheroids pretreated with *siHIST3H2A* or *siHIST3H2B*, followed by cisplatin treatment for 14 days. **C, D** siRNA efficiency in 3D spheroids after 14 days. Results represent the mean ± standard deviation of three experiments (**p* < 0.05, ***p* < 0.01).

**
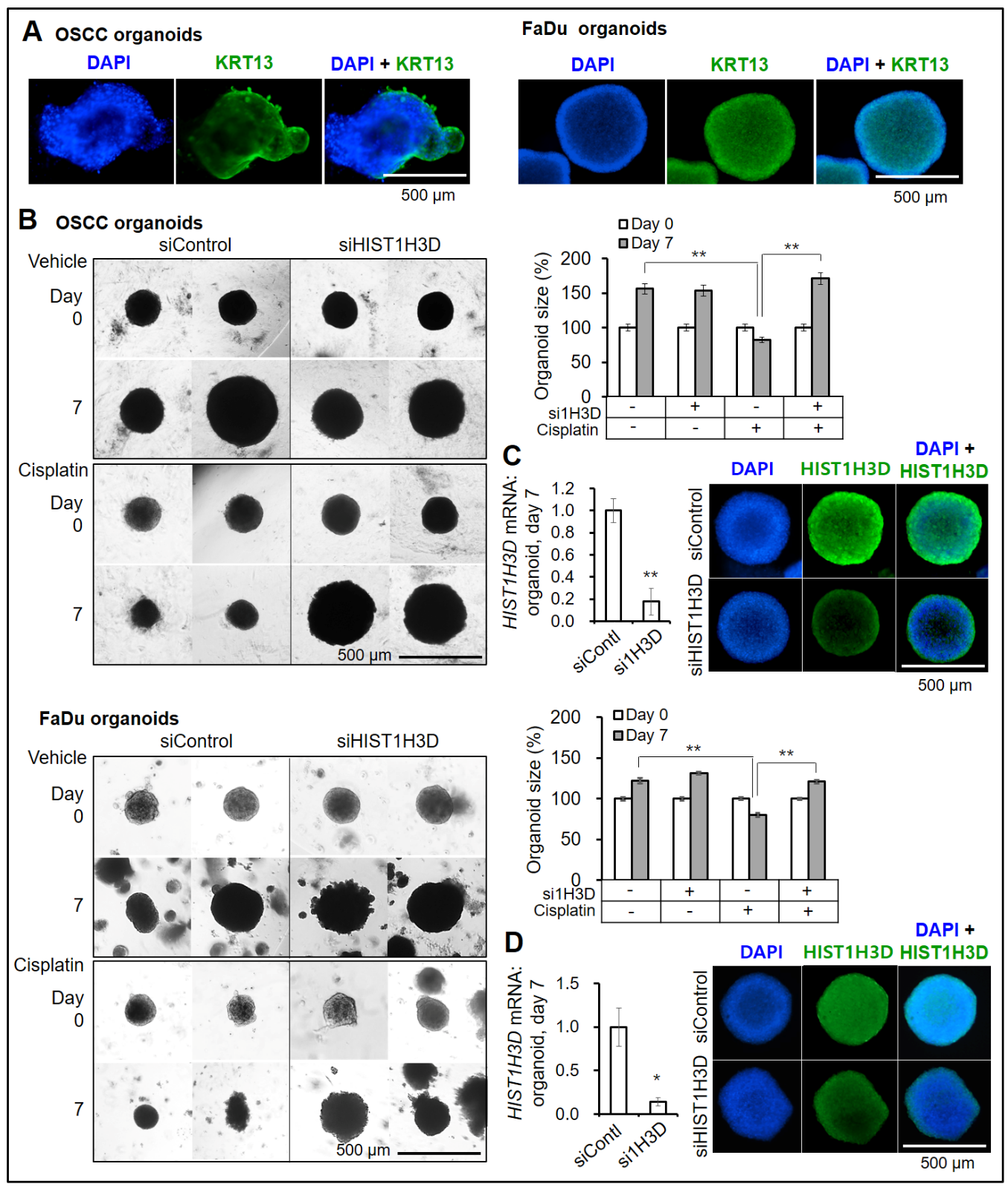
**

**Fig. S4 (related to Figure 2)**

**Effect of knockdown of *HIST1H3D* on the chemosensitivity in OSCC organoids.** Organids cultured with human OSCC tissue or FaDu xenografts were used. **A** IF staining for squamous epithelial marker *KTR13* of fixed OSCC organoids. **B** Representative images of organoids pretreated with *siHIST1H3D*, followed by cisplatin treatment for seven days. **C, D** *siHIST1H3D* efficiency in organoids using qPCR and IF staining. Results represent the mean ± standard deviation of three experiments (**p* < 0.05, ***p* < 0.01).


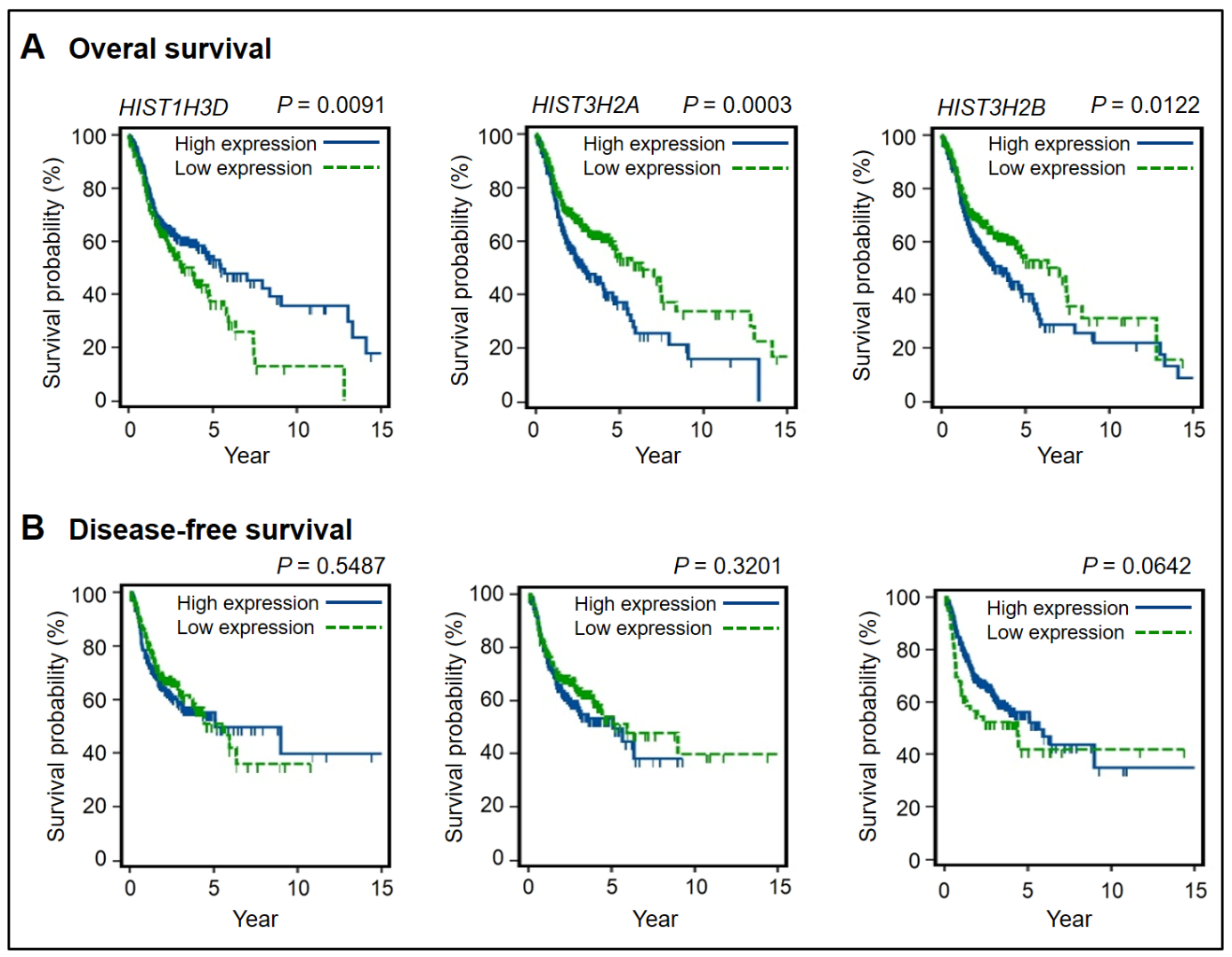


**Fig. S5 (related to Figure 4)**

**Effect of mRNA expression of selected histone protein genes on the survival of patients. A, B** Kaplan–Meier survival plots for *HIST1H3D*, *HIST3H2A*, and *HIST3H2B* generated with the TCGA dataset.

**
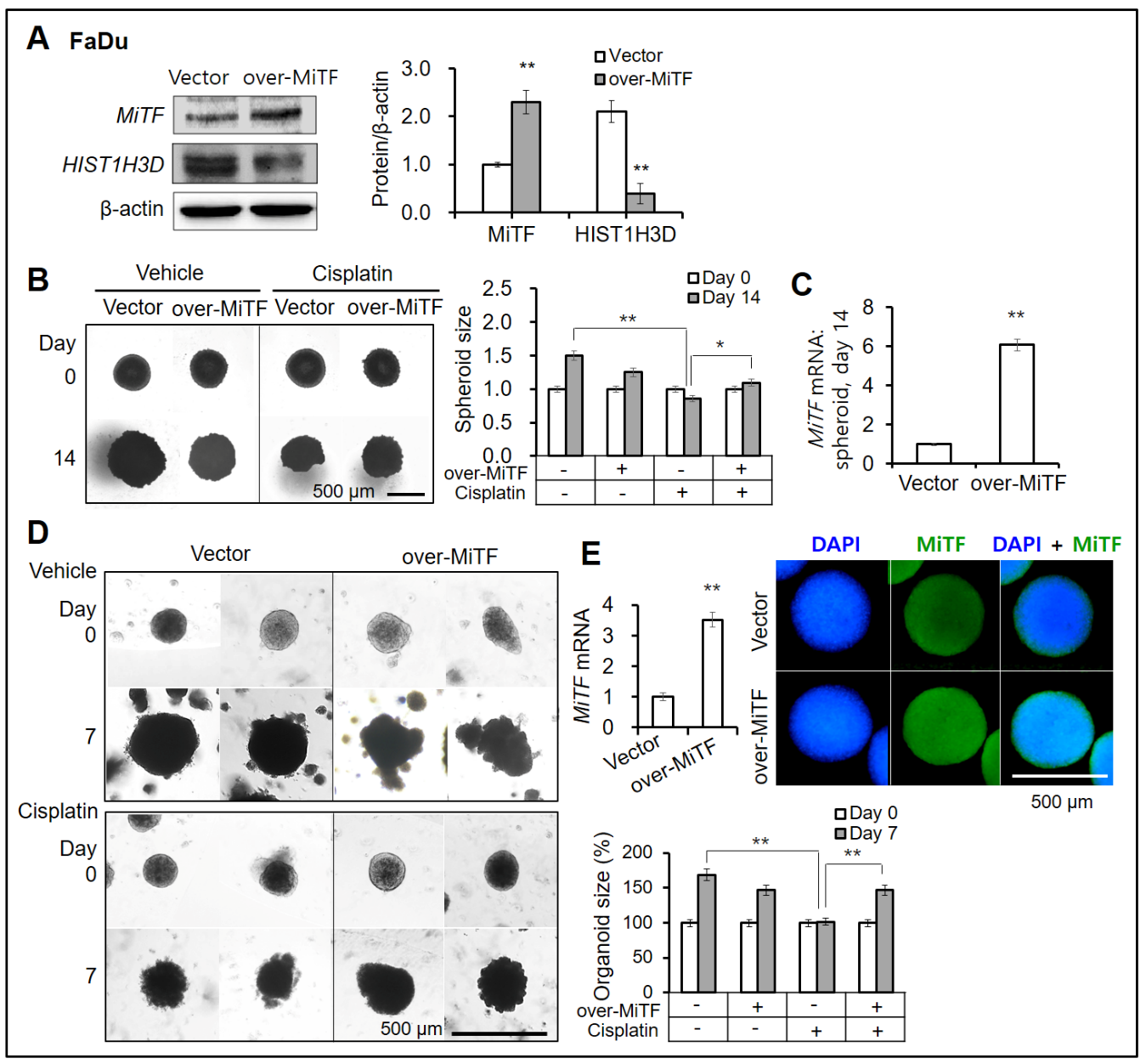
**

**Fig. S6 (related to Figure 5)**

**Effect of *MiTF* overexpression on the cisplatin efficacy in FaDu spheroids and organoids. A** *MiTF* overexpression-dependent decrease of *HIST1H3D* protein expression in FaDu cells. **B** Spheroids image pretreated with over-*MiTF*, followed by cisplatin treatment for 14 days. **C** *MiTF* mRNA expression showing siRNA efficiency in spheroids. **D** Organoids image pretreated with over-*MiTF*, followed by cisplatin treatment for seven days. **E** Overexpression efficiency in organoids after seven days of transfection analyzed by qPCR and IF staining. Results represent the mean ± standard deviation of three experiments (**p* < 0.05, ***p* < 0.01).

**
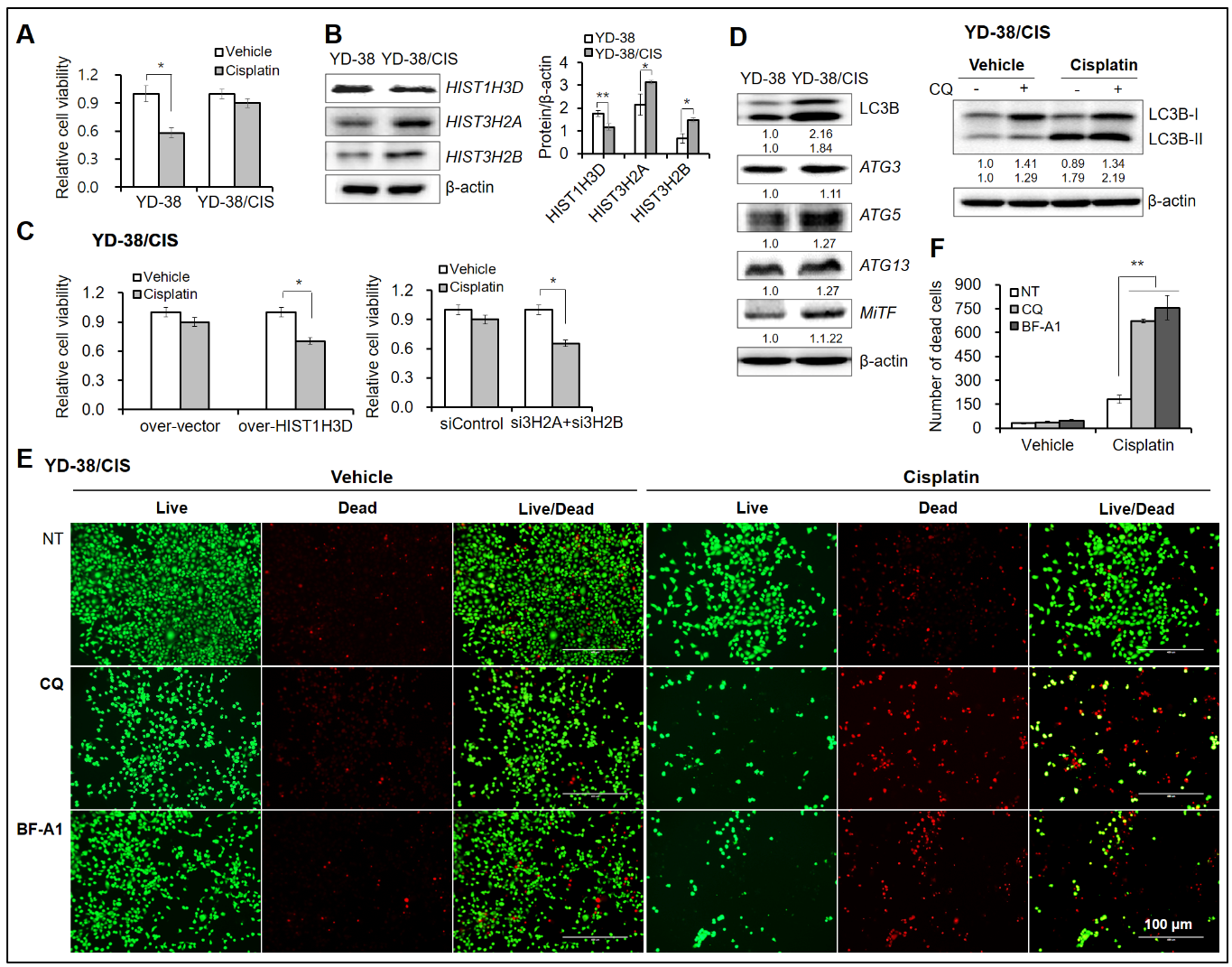
**

**Fig. S7 (related to Figure 6)**

**Effect of histone protein genes and autophagy inhibitors on cisplatin resistance in YD-38/CIS cells. A** Differential effect of cisplatin on cell viability between YD-38 and YD-38/CIS cells. **B** Histone protein expression level in both cell lines. **C** YD-38/CIS cell viability pretreated with *HIST1H3D*-overexpression vector or *siHIST3H2A* and *siHIST3H2B*, followed by cisplatin treatment. **D** Protein expression of autophagy-related genes. Autophagy status in YD-38/CIS pretreated with CQ by LC3B protein expression. The numbers below the western blot images indicate the relative intensities of each band normalized to the β-actin. **E** LIVE/DEAD staining of YD-38/CIS cells pretreated with autophagy inhibitors. **F** Dead cells were counted in each condition. Results represent the mean ± standard deviation of three experiments (**p* < 0.05, ***p* < 0.01).

**Supplementary Tables**

**Table S1. The forward and reverse primers for mRNA expression by qPCR**

| Gene name | F/R | sequence (5'→3') |
| --- | --- | --- |
| *HIST1H3D* | Forward | GTGTTCTGCCCAACATCCAG |
|  | Reverse | GTACGAGCCATTGCGAACTT |
| *HIST3H2A* | Forward | ATGTCCGGTCGTGGTAAGC |
|  | Reverse | GCTCCGAATAGTTGCCCTTG |
| *HIST3H2B* | Forward | ATGCCAGACCCGTCCAAATC |
|  | Reverse | TCTTGCCGTCCTTCTTCTGT |
| *NAP1L2* | Forward | TCCTGTGGAGGTTTGGTCTC |
|  | Reverse | CCAGCCTCTTCTTGACTGGA |
| *COL4A1* | Forward | AGGATTTACCGGACCACCAG |
|  | Reverse | TGGTCACCCTTGTCACCTTT |
| *CDH1* | Forward | CGACCCAACCCAAGAATCTA |
|  | Reverse | CTCCAAGAATCCCCAGAATG |
| *VIM* | Forward | GTTTCCAAGCCTGACCTCAC |
|  | Reverse | GCTTCAACGGCAAAGTTCTC |
| *TWIST1* | Forward | GCGCTGCGGAAGATCATC |
|  | Reverse | GCTTGAGGGTCTGAATCTTGCT |
| *ZEB1* | Forward | GCTTCTCACACTCTGGGTCT |
|  | Reverse | ACGTGCTCATTCGAGAGGAT |
| *CDH2* | Forward | GGTGGAGGAGAAGAAGACCAG |
|  | Reverse | GGCATCAGGCTCCACAGT |
| *ALDH1* | Forward | GCATTGTGTTAGCTGATGCCG |
|  | Reverse | AGAGAACACTGTGGGCTGGACA |
| *MiTF* | Forward | ACCGTCTCTCACTGGATTGG |
|  | Reverse | TGGGCTTGCTGTATGTGGTA |
| *GAPDH* | Forward | AGATCATCAGCAATGCCTCCTG |
|  | Reverse | CTGGGCAGGGCTTATTCCTTTTCT |

**Table S2. Clinicopathological parameters of OSCC tissues from patients used for histone protein genes immunostaining**

| Response to cisplatin | Age  Sex | Primary site | Differentiation | Lymph  node Meta | TMN stage | Stage |
| --- | --- | --- | --- | --- | --- | --- |
| S1 | 65  M | Buccal mucosa | Moderate | Y | pT3N2bM0 | III |
| S2 | 63  M | Lt. Mx. post. area | Moderate | N | cT3N0M0 | III |
| S3 | 74  F | Lt. Mn. post. area | Moderate | Y | pT3N2bM0 | III |
| S4 | 48  F | Buccal mucosa | Moderate | Y | pT1N1M0 | III |
| S5 | 56  M | Rt. Mn. post. area | Moderate | Y | pT4aN2bM1 | IVa |
| S6 | 82  F | Palatal | Poor | N | cT2N0M0 | II |
| R1 | 76  M | Buccal mucosa | Moderate | Y | pT4aN2bM0 | IVa |
| R2 | 59  F | Lt. Mn. post. area | Moderate | N | pT4aN0M0 | IVa |
| R3 | 53  M | Mouth floor | Well | Y | cT4aN2aM0 | IVa |
| R4 | 78  M | Lower lip | Moderate | Y | cT4N1M0 | IVa |
| R5 | 59  F | Lt. Mn.  post. area | Moderate | N | pT4aN0M0 | IVa |
| R6 | 64  M | Tongue | Well | Y | pT2N1M0 | III |

(c: clinical stage, p : pathologic stage)

**Table S3. The forward and reverse primers for ChIP, promoter luciferase assay, and chromatin accessibility assay**

| Experiments | | F/R | sequence (5'→3') |
| --- | --- | --- | --- |
| ChIP | 1 | Forward | TTAACACTGAATCGTGAAACTAACC |
|  |  | Reverse | TCTCTCCTGTATGTCTCAGGC |
|  | 2 | Forward | CAGCAGATTACACAAATACCAGTTG |
|  |  | Reverse | AATGTTGGCGCTGAAACTG |
|  | 3 | Forward | TGATCAAAGCCAGTTTCAGC |
|  |  | Reverse | TCTGTGCCATGTTTCTAGCTTT |
| Promoter Luciferase activity | | Forward | AATTTGGTACCCAGCAGATTACACAAATACCAG |
|  |  | Reverse | AATTTCTCGAGTCTGTGCCATGTTTCTAGC |
| Chromatin accessibility | *HIST1H3D* | Forward | AGCTTAACATCAGGTTTCATA |
|  |  | Reverse | TTTTAAAAAGACGCGCGC |
|  | *HIST3H2A* | Forward | GTTTGACCAACCCGAATTAAACA |
|  |  | Reverse | TGTGAGCTCCACCAGTGTTAAG |
|  | *HIST3H2B* | Forward | GCCAGCGAGCCCTTATGTAT |
|  |  | Reverse | TGTTCTACGTCATCGCTGGG |
|  | *ATG3* | Forward | CAGGCACGTTCAGTGTTACG |
|  |  | Reverse | GTCTGTCCTCGCTTTGCTTC |
|  | *ATG5* | Forward | CCCCAAGCCCCAATAACTAC |
|  |  | Reverse | GTGTTCTGCCTAACCCAGCA |
|  | *ATG13* | Forward | GGCTACCTGCGGTTCATCTC |
|  |  | Reverse | AAAGACGGAAAGGCGGTGTT |
|  | *MiTF* | Forward | GCCGAACTACAGATCCCAGG |
|  |  | Reverse | CCAGCGCGGGGAGTTTAC |

**References**

1. Choi SY, Oh SY, Kang SH, Kang SM, Kim J, Lee HJ *et al*. NAB 2-Expressing Cancer-Associated Fibroblast Promotes HNSCC Progression. Cancers (Basel). 2019; 11: 388.

2. Kang SH, Oh SY, Lee HJ, Kwon TG, Kim JW, Lee ST *et al*. Cancer-Associated Fibroblast Subgroups Showing Differential Promoting Effect on HNSCC Progression. Cancers (Basel). 2021; 13: 654.

3. Kim J, Kang SM, Oh SY, Kang SH, Lee I, Hwang JC *et al*. Early Growth Response 1-Dependent Downregulation of Matrix Metalloproteinase 9 and Mouse Double Minute 2 Attenuates Head and Neck Squamous Cell Carcinoma Metastasis. Cell Physiol Biochem. 2018; 50: 1869-1881.
