# Supplementary figures and images for: Chromatin remodeling-driven autophagy activation induces cisplatin resistance in oral squamous cell carcinoma
Source: Cell Death Dis. 2024 Aug 13;15(8):589. doi: 10.1038/s41419-024-06975-1 (PMC11322550; doi:10.1038/s41419-024-06975-1)

Fig. S1B

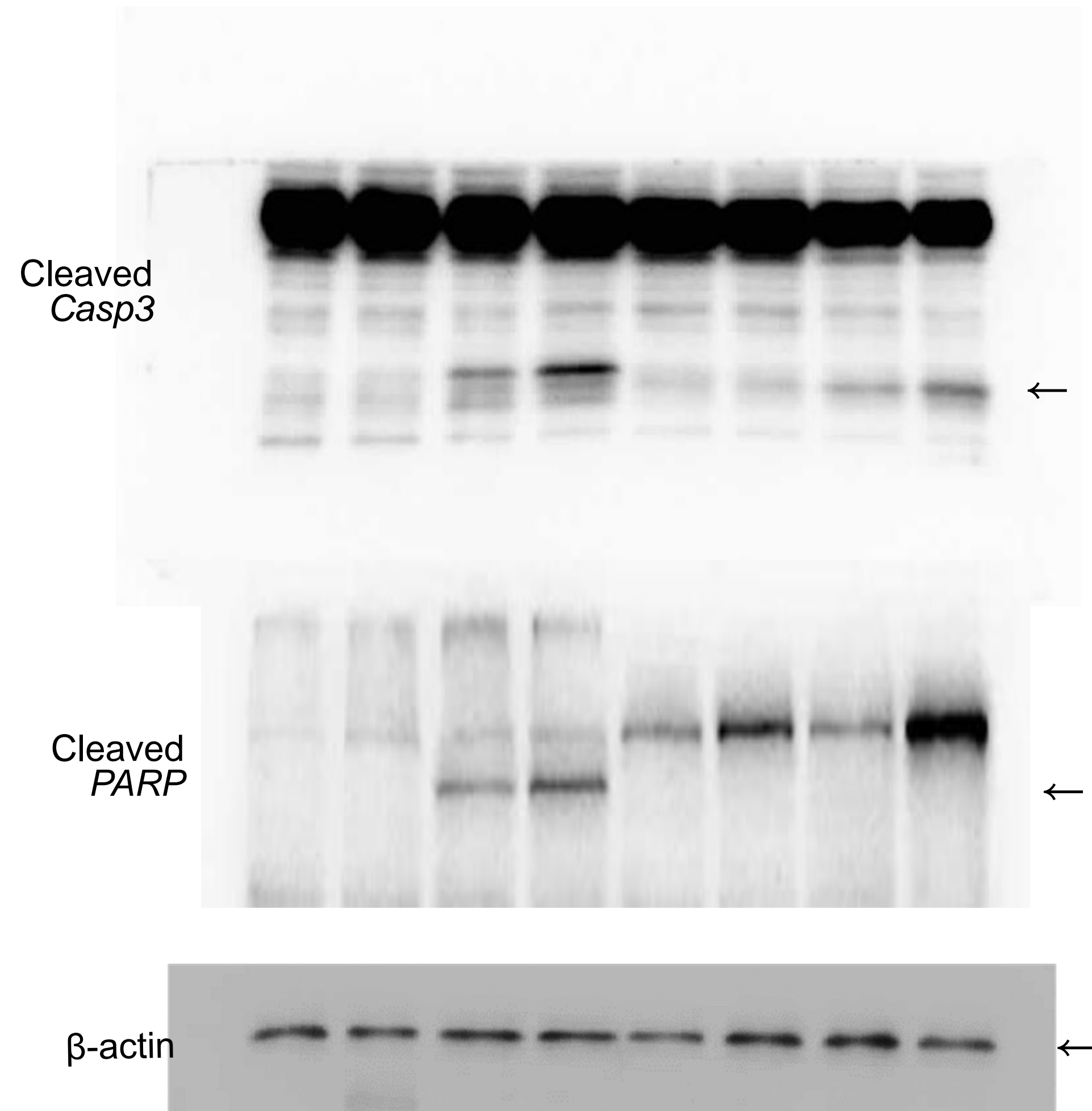

Fig. 1C

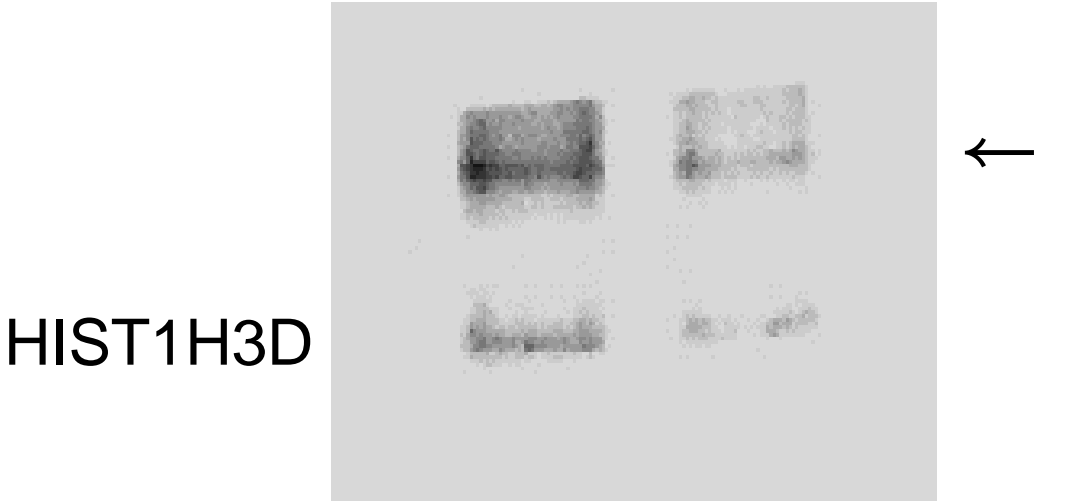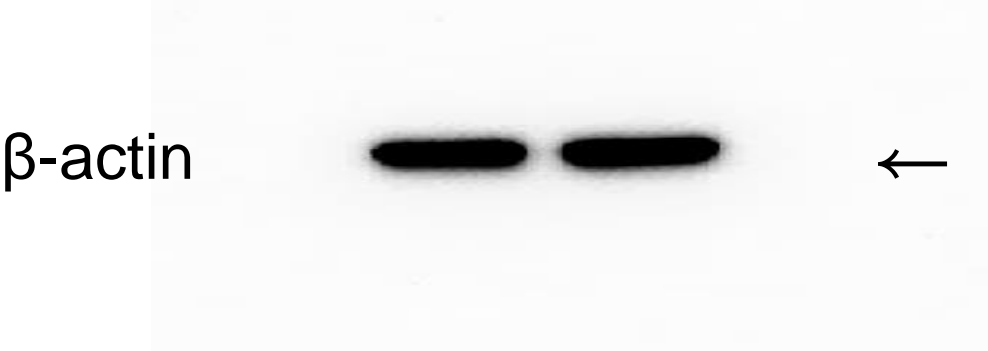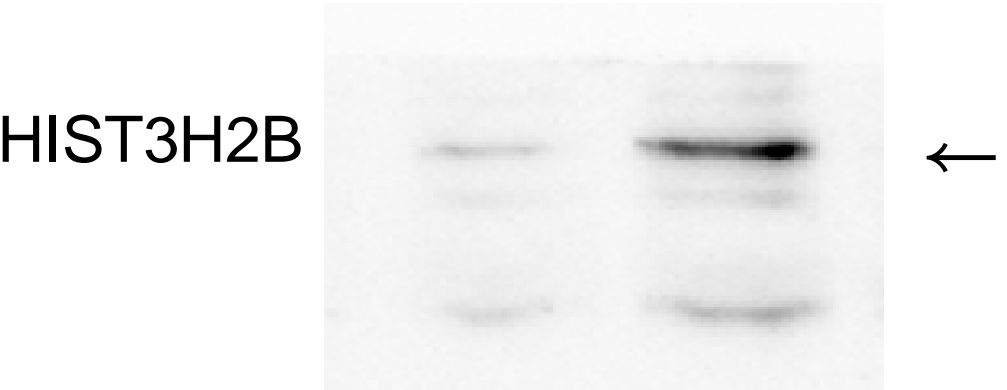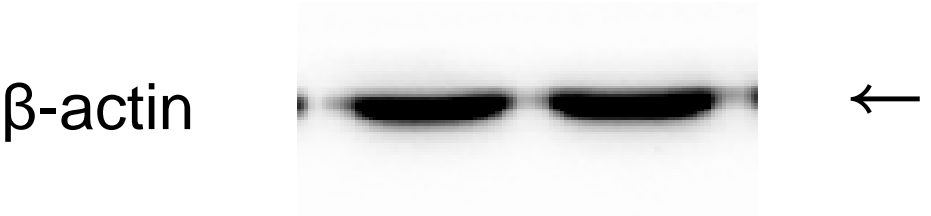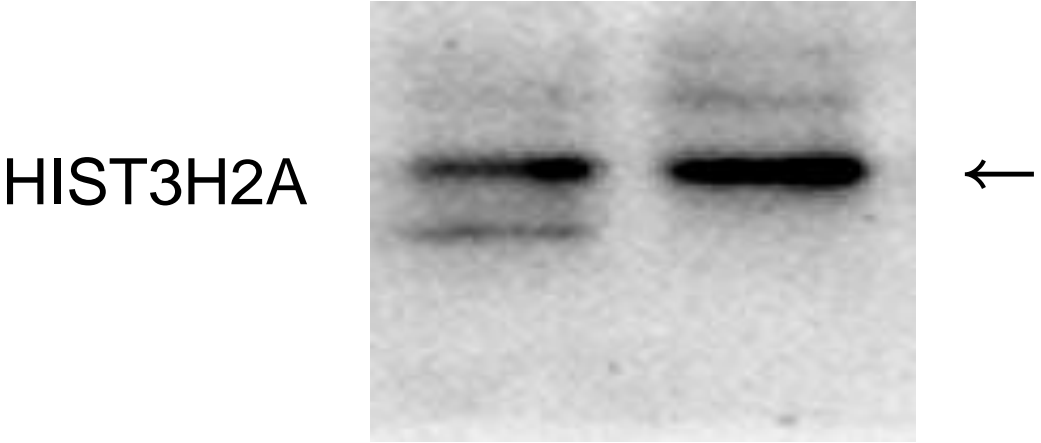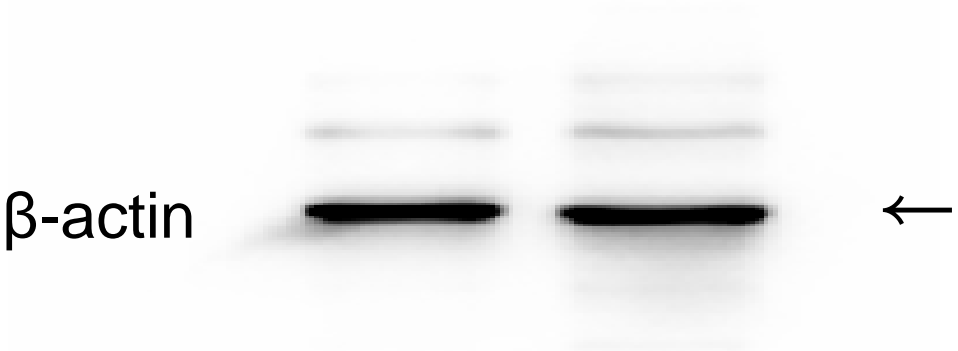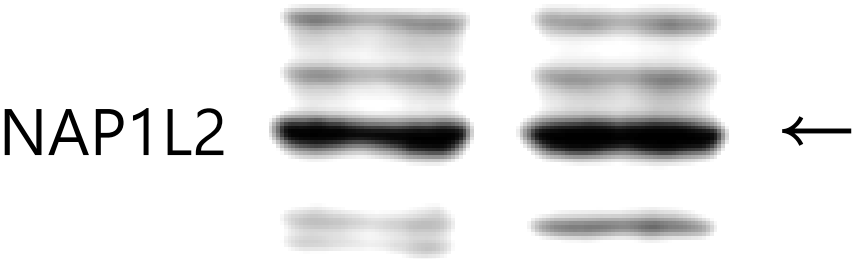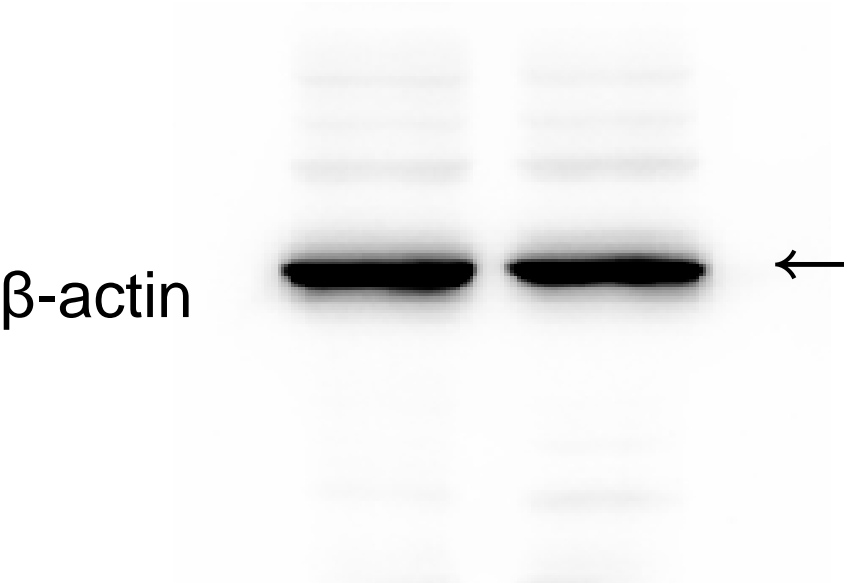

Fig. 5A

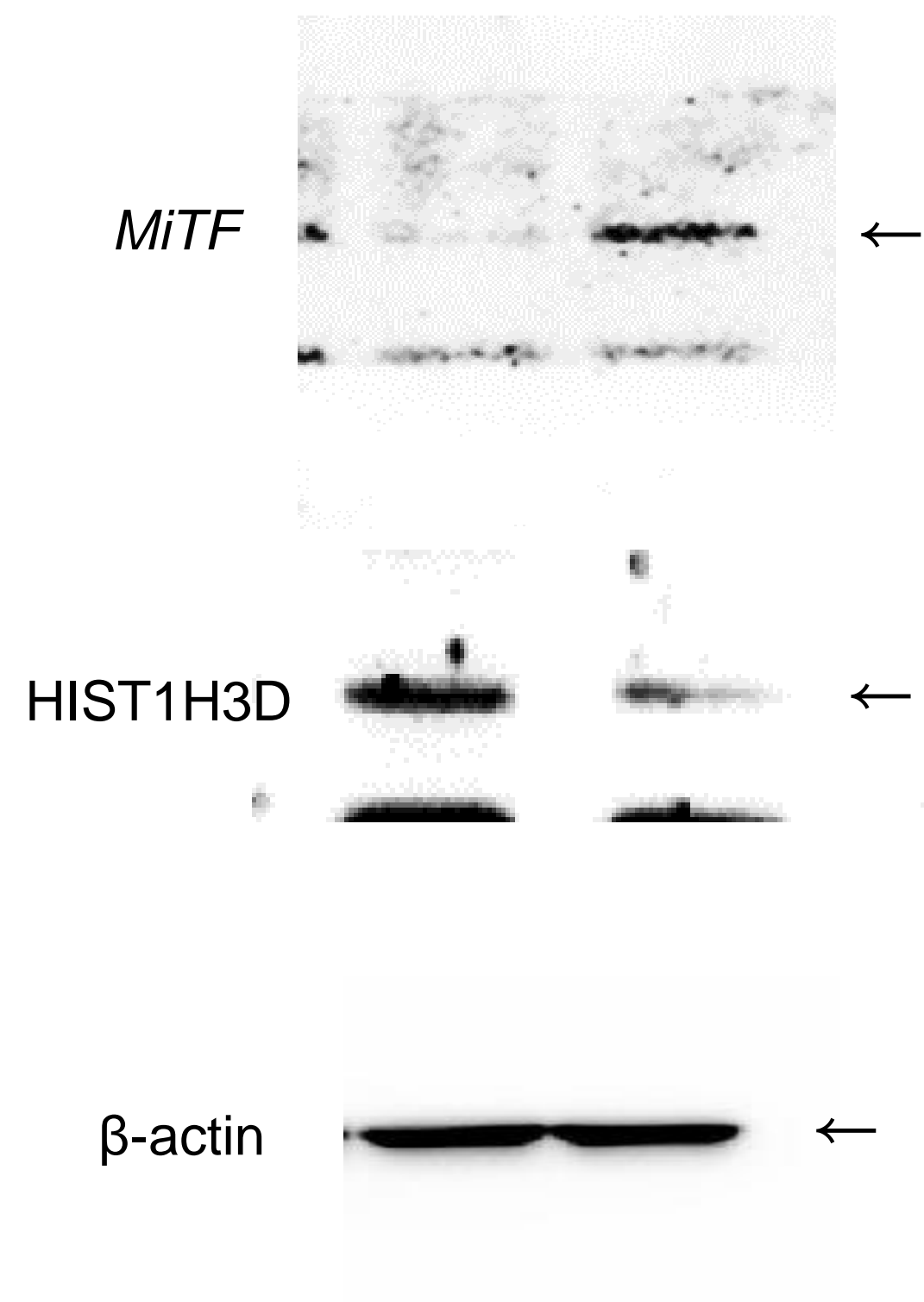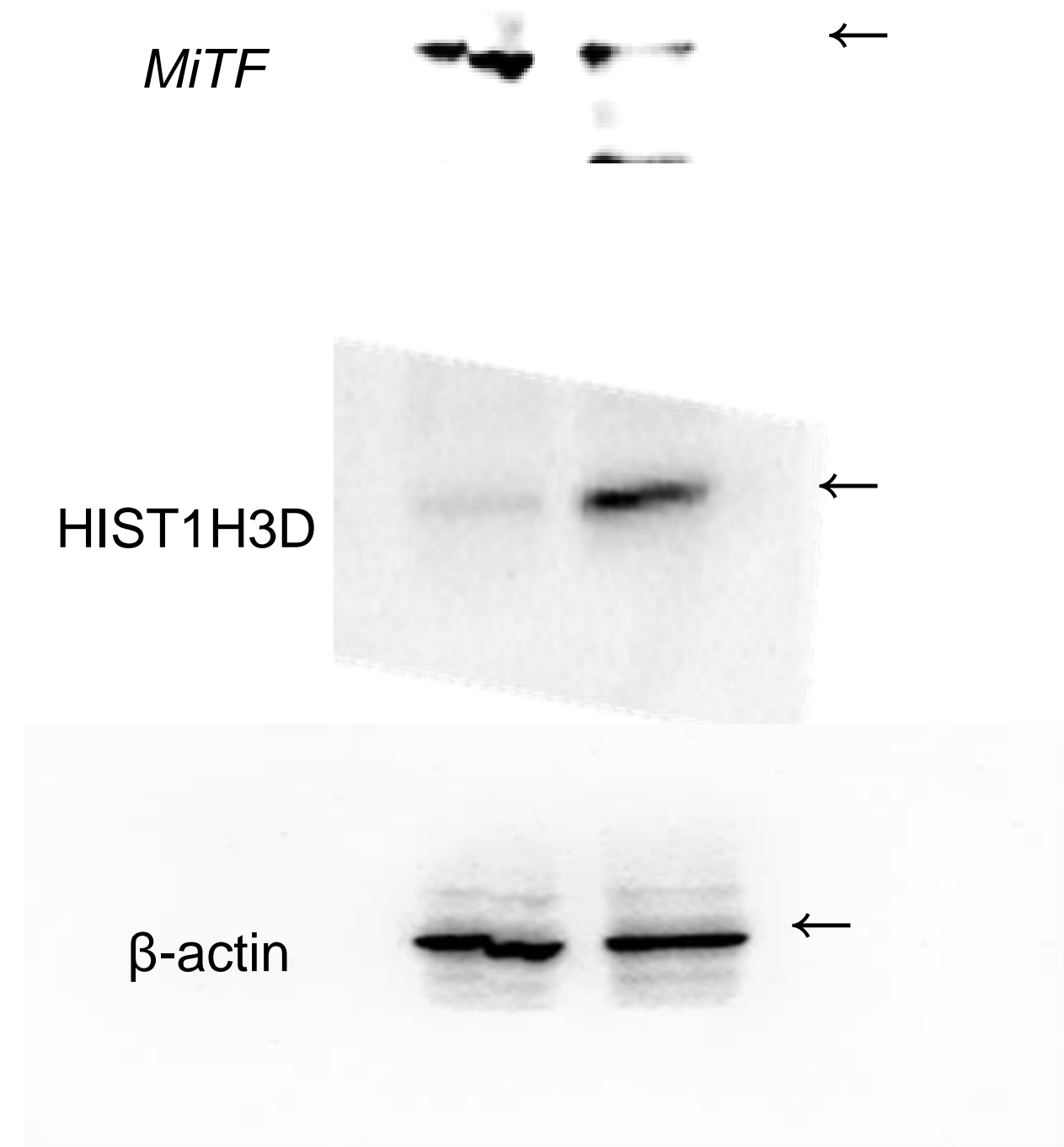

Fig. S6

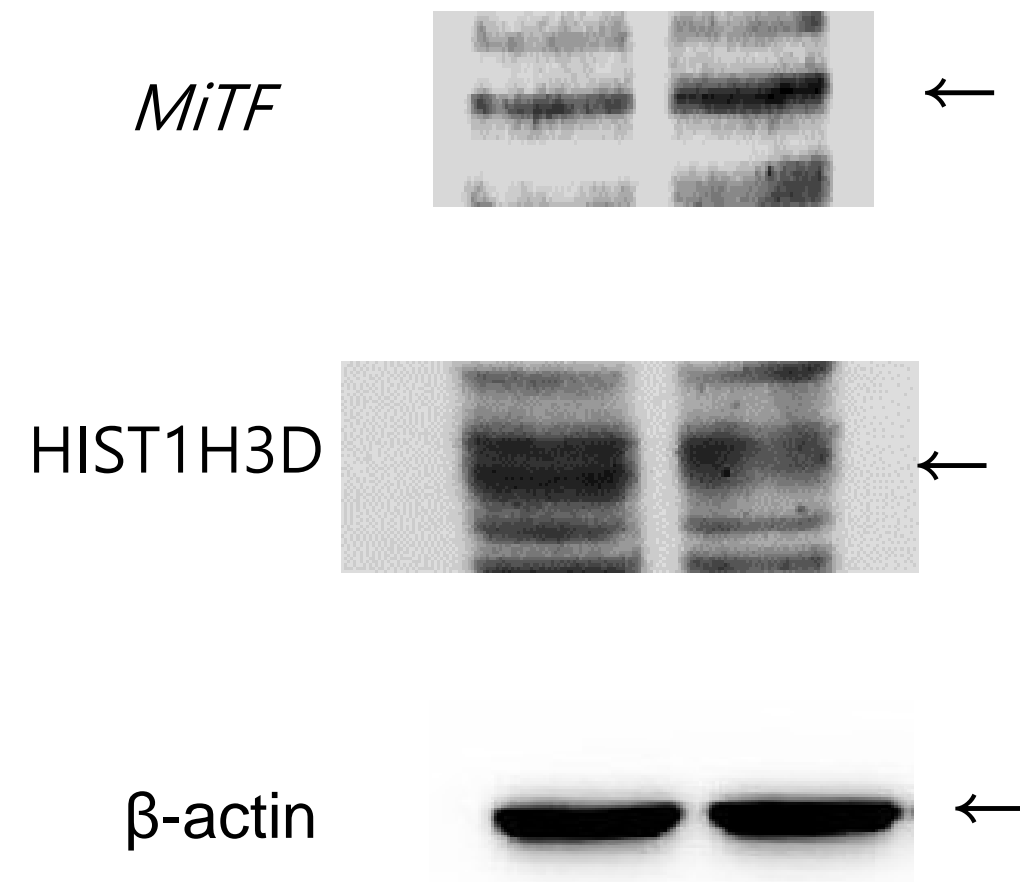

Fig. 6

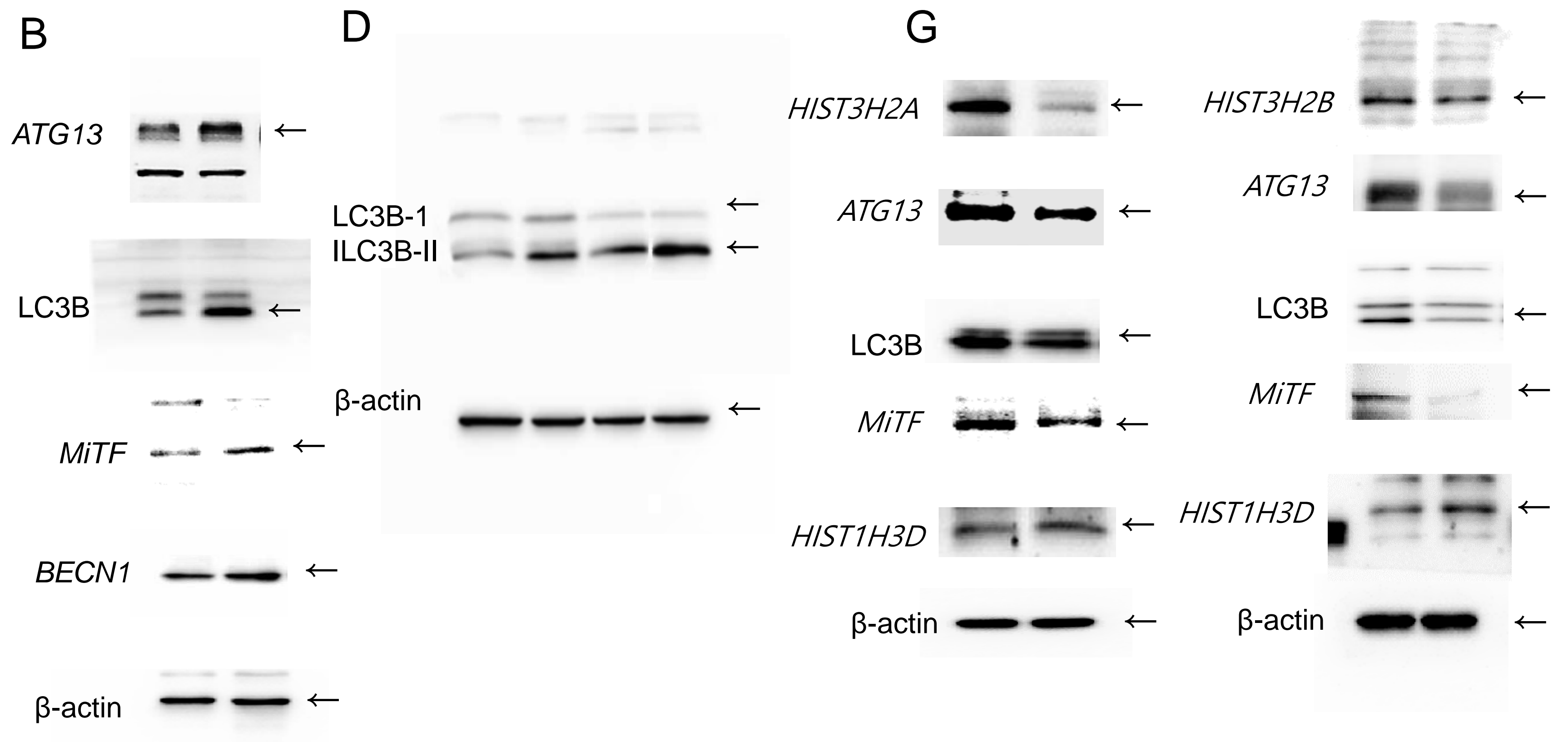

Fig. S7

B

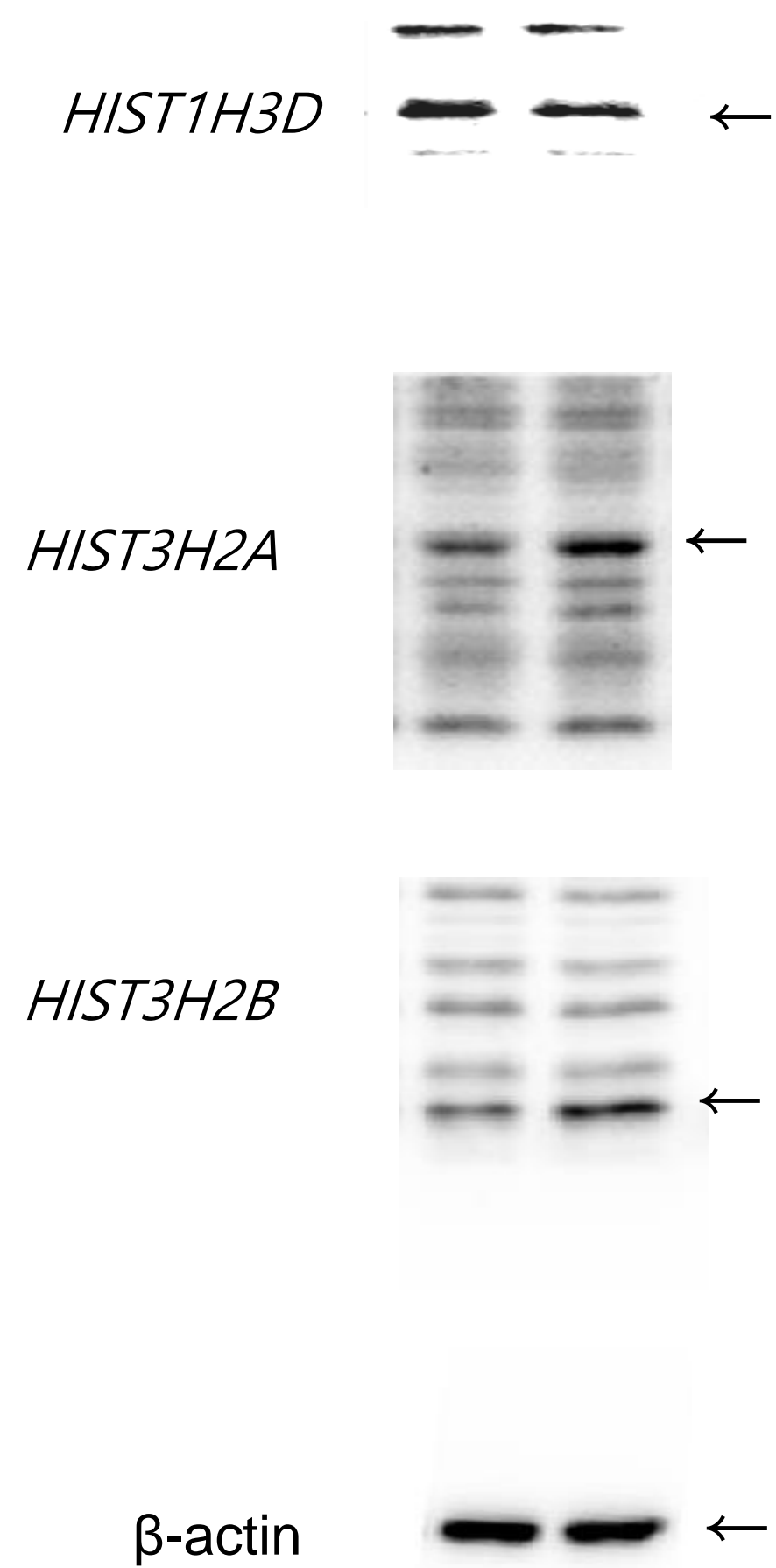

D

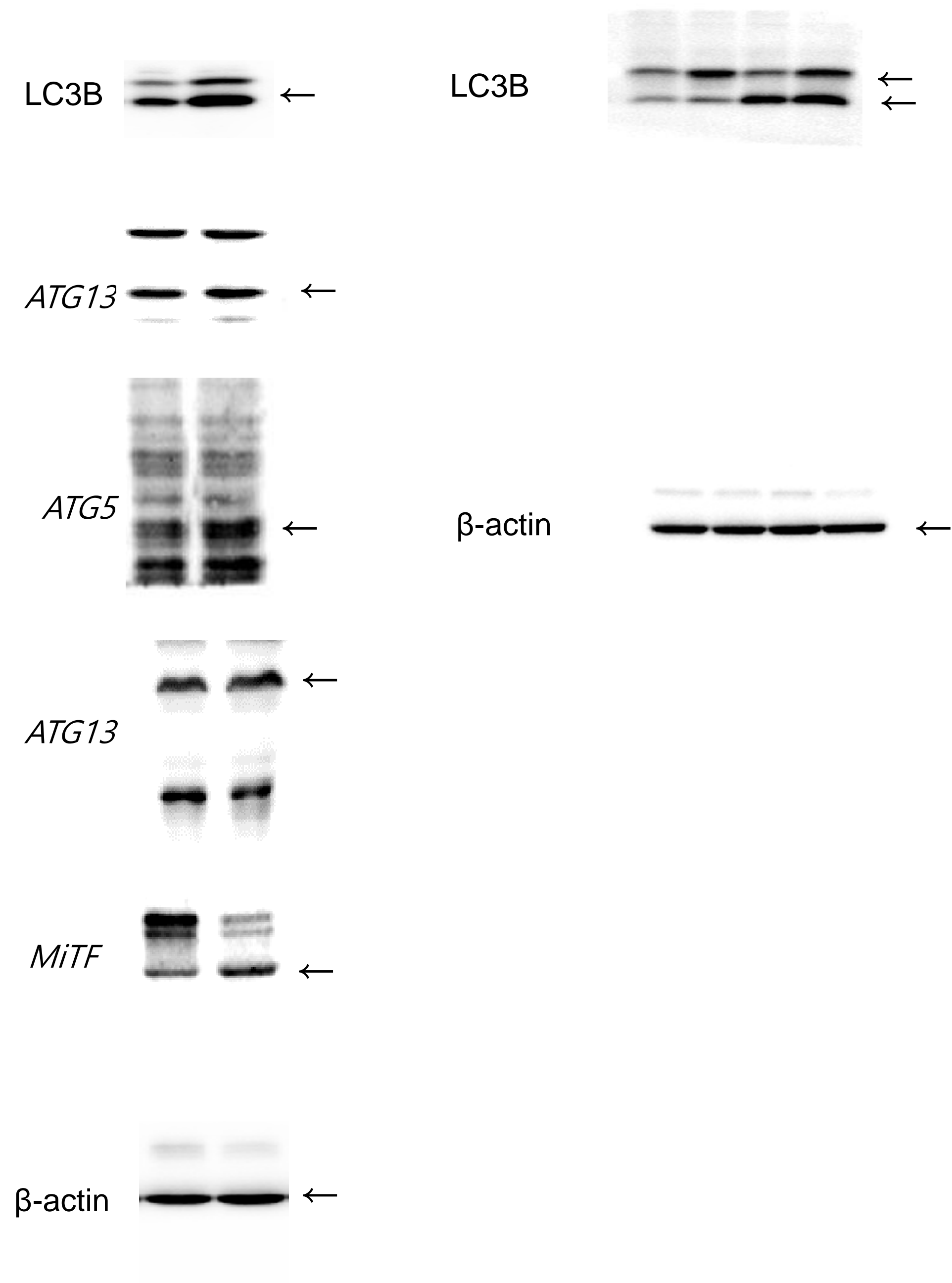

Supplement: Supplementary file 2 — Western raw data [file 41419_2024_6975_MOESM2_ESM.pdf]
